# Whole genome duplication drives transcriptome reprogramming in response to drought in alfalfa

**DOI:** 10.1007/s00299-025-03593-9

**Published:** 2025-09-09

**Authors:** D. F. Santoro, A. W. Anderson, S. N. Alavi, V. A. Malatesta Pierleoni, D. Rosellini

**Affiliations:** 1 Department of Agricultural, Food and Environmental Sciences, University of Perugia, Borgo XX Giugno 74, 06121 Perugia, Italy; 2 Interuniversity Consortium for Biotechnology (CIB), Area Science Park, Padriciano 99, 34149 Trieste, Italy

**Keywords:** Gene co-expression network, *Medicago sativa*, Photosynthesis, RNA-Seq, Sexual polyploidization, Water deficit, WGCNA

## Abstract

**Key message:**

Genome doubling did not enhance drought tolerance in alfalfa, but may set the stage for long-term adaptation to drought through a novel transcriptional landscape.

**Abstract:**

Whole genome duplication (WGD) has been shown to enhance stress tolerance in plants. Cultivated alfalfa is autotetraploid, but diploid wild relatives are important sources of genetic variation for breeding. Investigating how WGD affects gene expression in stress conditions could provide better understanding for use of diploid genetic resources. In this work, we compared the drought response of neotetraploid plants obtained by bilateral sexual polyploidization with diploid full sibs, by measuring physiological and biochemical traits and RNA-seq. Without drought, 4x plants had lower photosynthetic potential than 2x plants per unit leaf area, but larger leaves allowed them to outperform the per leaf photosynthetic potential of 2x plants. Physiological and biochemical traits were significantly affected by drought in both 2x and 4x plants, but the differences between ploidies were small and nonsignificant. Proline levels were higher in 4x than 2x plants, both in control and drought conditions, indicating that larger cells with higher volume-to-surface ratio of 4x  plants require a higher osmolyte concentration. RNA-seq and gene network analyses showed that more genes were affected by drought at 4x than at 2x level, with downregulation of hundreds of genes involved in photosynthesis and stomatal movement at 4x level, suggesting that WGD made the 4x plants more responsive to drought. Genes involved in proline, phytormone and cell wall functions were also transcriptionally affected by drought in 4x plants. We conclude that WGD did not immediately enhance drought tolerance in alfalfa, but may set the stage for long-term adaptation to drought through a novel transcriptional landscape.

**Supplementary Information:**

The online version contains supplementary material available at 10.1007/s00299-025-03593-9.

## Introduction

Polyploidy, defined as the presence of more than two sets of homologous chromosomes in a nucleus, represents a major driving force of genome evolution and speciation in angiosperms (Wood et al. 2009; Madlung 2013; Van De Peer et al. 2017). Research has shown that polyploidy has played a crucial role in the domestication of many crop species by enhancing their genetic diversity and adaptability (Salman-Minkov et al. 2016). Polyploid organisms arise through whole-genome duplication (WGD) within a species (autopolyploidy) or involving hybridisation between related species (allopolyploidy). WGD often involves structural genome changes (Fawcett et al. 2009; De Storme and Mason 2014; Vanneste et al. 2014), that can result in unique physiological and morphological characteristics not found in the diploid progenitors (Heslop-Harrison et al. 2023). Polyploidization increases cell size, leading to a complex network of interactions within the cell, from chromatin structure and gene regulation to the function of organelles and the movement of molecules (Doyle and Coate 2019; Soltis and Soltis 2012).

Polyploidy has often been shown to enhance tolerance to stress conditions such as drought, salinity, and extreme temperatures through mechanisms such as genome buffering, increased gene expression flexibility, and epigenetic modifications (Van de Peer et al. 2021; Tossi et al. 2022). This adaptability is thought to provide an evolutionary advantage during shifting environmental conditions, as seen in the increased endopolyploidy in plant tissues experiencing stress, enabling resilience and recovery through cellular redundancy and genomic plasticity (Morris et al. 2024).

Drought is a major challenge for global agriculture, with severe adverse effects on global crop productivity, exacerbated by climate change (Dai 2013; Touma et al. 2015; Schwalm et al. 2017). While the definition of drought is a matter of debate (Tardieu et al. 2018), in the context of agriculture, one component of drought is water deficit, defined as available water not meeting plant evapotranspiration needs.

Polyploids have shown enhanced tolerance to drought compared to their diploid counterparts through larger cell sizes, modified root-to-shoot ratios, and improved water-use efficiency (Maherali et al. 2009; Tossi et al. 2022). This tolerance is partially attributed to larger xylem vessels having increased hydraulic conductivity, enhanced antioxidant capacity to combat oxidative stress, better regulation of osmolyte content and pre-activated abscisic acid (ABA) responses (Del Pozo and Ramirez-Parra 2014; Yang et al. 2014; Zhang et al. 2015; Ruiz et al. 2020).

Despite the widespread occurrence of polyploids under drought conditions (Te Beest et al. 2012; Hao et al. 2013; Folk et al. 2020; Van de Peer et al. 2021; Tossi et al. 2022), there are still gaps in our understanding of polyploidy advantage at the physiological, molecular and particularly the gene expression levels. With the advancement of sequencing technologies, it is becoming feasible to further our understanding of how polyploids truly differ from their diploid progenitors. One specific tool for this study is RNA sequencing (RNA-Seq), which allows for detailed studies across many plant species and transcriptomic insight into the functionality of polyploid genomes.

Cultivated alfalfa (*Medicago sativa* L., 2n = 4x = 32) is one of the most widespread perennial forage legumes globally, valued for its nutrient-rich feed and versatility for hay, pasture, and silage production across a wide range of environmental conditions. While comparatively alfalfa is generally considered a drought tolerant plant, thanks to its deep root system (Abdul-Jabbar et al. 1982), prolonged and severe drought leads to loss of yield and undesirable forage quality (Carter and Sheaffer 1983; Peterson et al. 1992). Sustaining and increasing production under drought is currently a major focus of many alfalfa breeding programs. Many traits associated with drought tolerance have been and are continually being investigated (Singer et al. 2018; Diatta et al. 2021; Kang et al. 2023; Zhou et al. 2024). Alfalfa varieties considered drought tolerant have been identified with increased accumulation of malondialdehyde (MDA) as well as other antioxidants, which is in line with studies in soybean, wheat, and maize (Zhang et al. 2019). Efforts are currently being made to characterize diverse wild relatives, both diploid and polyploid, within the *M. sativa* complex to identify sources of drought-tolerance germplasm for breeding programs (Ray et al. 2015; Humphries et al. 2021).

In this work, we have investigated the effects of WGD on drought tolerance in alfalfa, by comparing physiological traits and transcriptomes of full-sib diploid and neotetraploid plants. We found that, although the phenotypic differences between diploids and tetraploids were limited, WGD resulted in a distinctive transcriptional response to drought. We identified genes, pathways and hubs that could provide a polyploid advantage and can be targets of breeding programs for improved yields under drought.

## Materials and methods

### Plant material and experimental conditions

The plants used in this work were previously described by (Rosellini et al. 2016), and consisted of three diploid, S8, S16 and S24, and three tetraploid, S29, S48 and S60, genotypes obtained through bilateral sexual polyploidization from crossing two diploid meiotic mutants, PG-F9 and 12P, (Tavoletti 1994; Barcaccia et al. 1997; Tavoletti et al. 1991). The genotypes have been maintained at the Department of Agricultural Food and Environmental Sciences of the University of Perugia through yearly cloning by stem cuttings. The study was conducted in a climate-controlled growth chamber at 23 ± 1 °C and 80% humidity with light levels of 324 µmol m^−2^ s^−1^ provided by fluorescent tubes and halogen bulbs, with 16/8 photoperiod. The pots were arranged in a randomized block design, containing soil, sand and peat moss at a ratio of 2:1:1 with five blocks, each containing three rows (control, stress and recovery). During the 2-week acclimatization period, irrigation was provided to meet evapotranspiration need with tap water.

### Stress treatment

**Fig. 1:**
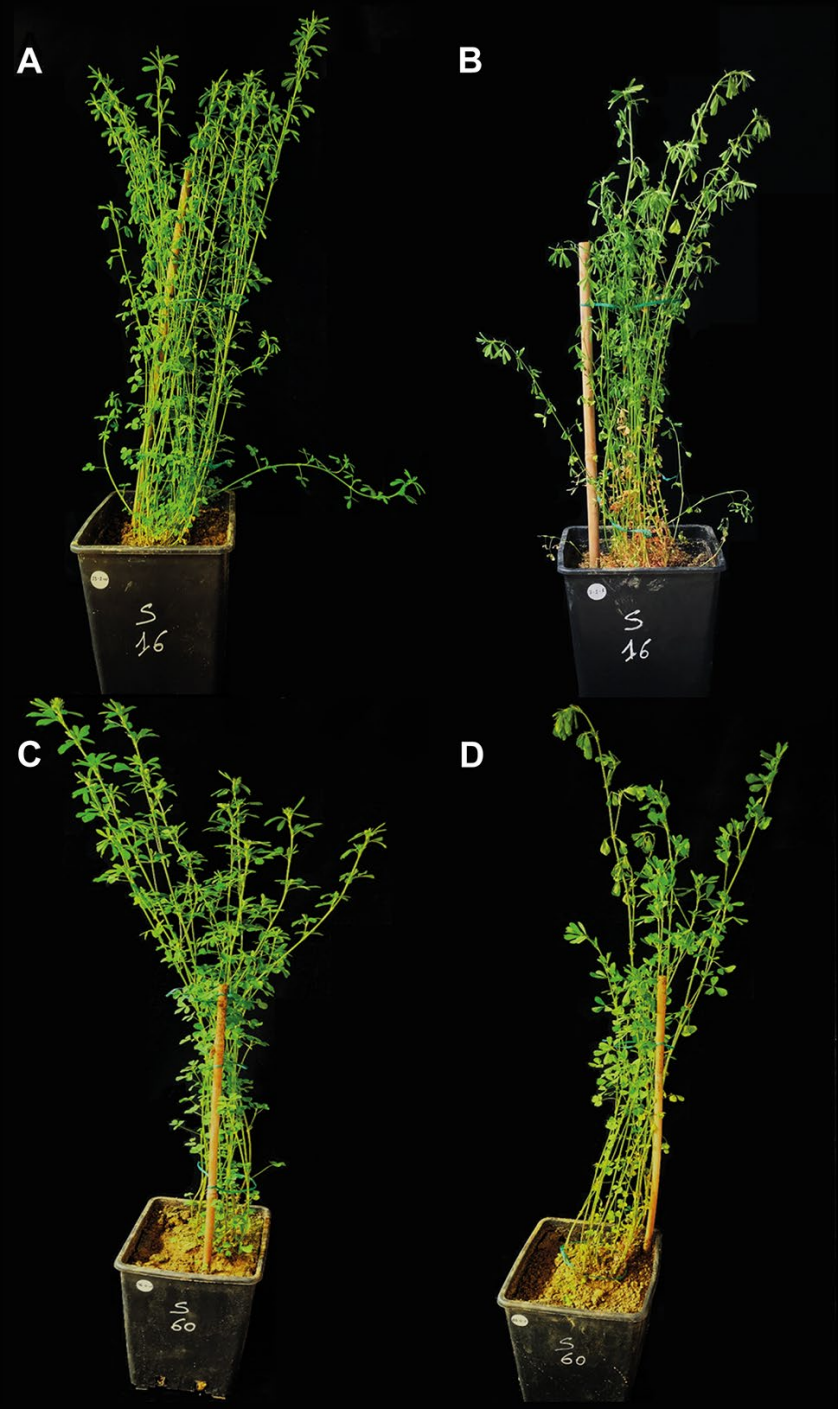
Phenotype of diploid S16 (**A**, **B**) and tetraploid S60 (**C**, **D**) genotypes under control (**A**, **C**) and drought conditions (9.5% soil water content, **B**, **D**)

To initiate the study, all plants were irrigated to field capacity and assigned one of two watering regimes: well-watered and not watered (drought). To identify the soil water content at which stress signs became evident, a preliminary trial was conducted with one plant per genotype. Soil volumetric water content was checked daily in the two conditions using a Terrasense device (Netsens), which uses frequency domain reflectometry to determine the dielectric constant. The average of three measurements per pot was used as the daily water content value. Pots were also weighed throughout the preliminary experiment to verify device accuracy. Wilting of shoot tips (Fig. 1) was observed in all genotypes below 10% soil water content. A stress threshold of 9.5% ± 0.2% was chosen for drought stress measurements. Three water regimes were applied. Control plants were watered every 2–3 days to maintain optimal water status. Drought treatment was applied by suspending irrigation until the threshold soil water content was reached, and data were collected. Severe drought treatment was applied by allowing plants to wilt severely to complete loss of leaf turgidity before being re-watered to visually assess their recovery after 48 h. No physiological or biochemical data were collected on the latter group of plants.

### Physiological parameters

Gas-exchange-based data were collected using a Li-Cor 6400 instrument equipped with a 3 × 2 cm chamber: photosynthetic rate (µmol CO_2_ m^−2^ s^−1^), stomatal conductance (mol H_2_O m^−2^ s^−1^), transpiration rate (mmol H_2_O m^−2^ s^−1^), and intercellular CO_2_ concentration (µmol CO_2_ mol^−1^), for one measurement per trifoliate. Parameters were set at: Lux = 1000 (Units), reference CO_*2*_ = 400, flow = 500. Three young fully expanded leaves per plant were individually placed in the chamber for 1 min immediately after removal from the plant. Chlorophyll content was estimated using a SPAD device (Minolta) twice on each trifoliate leaf. Data were collected at 2 time points in this study. Baseline data was collected after both control and treated plants were watered to saturation and allowed to acclimate (no stress/ day zero). Stress time point data were collected from stressed and control plants when stressed plants reached the soil water content threshold defined above.

### Sample Collection

Multiple young fully expanded non-senescing trifoliate leaves were harvested at stress time point from each alfalfa plant, immediately frozen with liquid nitrogen and stored at − 80 °C until further processing. A total of thirty-six leaf samples (6 genotypes × 2 treatments × 3 biological replicates) were used in the following molecular techniques.

### Biochemical characterization

Proline content was determined according to (Carillo and Gibon 2011). For this study, 50 mg of flash-frozen leaf tissue was homogenized in liquid nitrogen and extracted with 40% ethanol. A volume of 125 µL of the extract was mixed with 1 mL of the reaction mix containing 1% (*w*/*v*) ninhydrin solubilized in 60% (*v*/*v*) acetic acid and 20% (*v*/*v*) ethanol. The mixture was incubated at 95 °C for 20 min in the dark and centrifuged at 10,000 rpm for 1 min at room temperature in a table microfuge. The absorbance of the supernatant was then measured at 520 nm. Quantification was performed using an external calibration curve prepared using proline solutions at concentrations ranging from 0.02 to 5 mmol. Lipid peroxidation was measured using a modified thiobarbituric acid (TBA) method (Dhindsa et al. 1981). Fifty mg of flash-frozen leaf tissue was homogenized in 1 ml 0.1% (*w*/*v*) TCA solution. The homogenate was centrifuged at 10,000 rpm for 5 min and 0.5 ml of the supernatant was added to 1 ml 0.5% (*w*/*v*) TBA in 20% TCA. The mixture was incubated in a 95 °C water bath for 30 min, and the reaction stopped by placing the reaction tubes in an ice bath. Then the samples were centrifuged at 10,000× *g* for 10 min, and the absorbance of the supernatant was read at 532 nm. The value for non-specific absorption at 600 nm was subtracted. The amount of malondialdehyde (MDA) was calculated using its extinction coefficient of 155 mM^−1^ cm^−1^.

### RNA extraction

Total RNA was isolated from 60 mg of ground flash-frozen tissue using the RNeasy Plant Mini Kit (Qiagen, Germantown, MD, USA) with degradation and contamination evaluated by 1% agarose gel electrophoresis. Purity and concentration were checked using the NanoDrop spectrophotometer (Thermo Fischer Scientific, Waltham, MA, USA). Total RNA quantity was determined with a Qubit® 2.0 Fluorometer using the Qubit® RNA BR assay kit (Invitrogen™). Integrity was assessed using the Agilent Bioanalyzer 2100 system (Agilent Technologies, Santa Clara, CA, USA). Samples with 260/280 and 260/230 ratios ranging from 1.8 to 2.2 were used for sequencing. The quality was assessed by the RNA integrity number measurement. Our average of 6.6 indicated samples were suitable for sequencing.

### Library preparation for RNA-seq

The transcriptome sequencing was conducted by Novogene (Cambridge, UK). Libraries were generated on the Illumina Hiseq2500 (150 bp paired-end reads; 6 G) using the NGS RNA Library Prep Set (Novogene Biotech, PT044). Libraries were sequenced on the Illumina HiSeq2500 platform to generate paired-end 2 × 150 bp reads. Raw reads were first processed through in-house Perl scripts to remove reads containing adapters or poly-N, and low-quality reads. Q20, Q30, GC content and sequence duplication levels of the clean data were calculated. All the downstream analyses were based on high-quality clean data (Supplementary Table S1).

### De novo assembly for alfalfa leaf transcriptome reconstruction and gene function annotation

De novo transcriptome assembly was performed using Trinity (2.6.6 version) with min_Kmer_Cov = 3 and min_glue = 4 (Grabherr et al. 2011). Corset (1.09 version,—m 10)was used to hierarchically groups transcripts by sequence similarity and expression (Davidson and Oshlack 2014). The longest transcript from each cluster was then designated as a unigene, which refers to a single representative transcript per gene locus, encompassing all associated isoforms and transcript variants. Benchmarking Universal Single-Copy Orthologs (BUSCO software, 3.0.2 version) was used for assembly assessment and gene prediction (Simão et al. 2015). Unigene functional annotations were obtained through: National Centre for Biotechnology Information (NCBI), non-redundant protein sequences (Nr, Diamond software, 2.1.6 version, *e*-value threshold 1e−5) (Buchfink et al. 2014), NCBI non-redundant nucleotide sequences (Nt, NCBI blast software, 2.9.0 version, *e*-value threshold 1e−5), Protein family (Pfam, hmmscan software, HMMER 3.1 version, *e*-value threshold 0.01) (Finn et al. 2011), Cluster of Orthologous Groups of Proteins (KOG/ COG, Diamond software, 2.1.6 version, *e*-value threshold 1e−5) (Buchfink et al. 2014), Swiss-Prot (Diamond software, 2.1.6 version, *e*-value threshold 1e−5), Kyoto Encyclopaedia of Genes and Genome (KEGG, Diamond and KAAS software, 2.1.6 version, *e*-value threshold 1e−5) (Buchfink et al. 2014; Moriya et al. 2007) and GO (blast2GO software, b2g4pipe_ v2.5 version, *e*-value threshold 1e−6) (Götz et al. 2008). The *M. sativa* transcriptome was published in NCBI-SRA (https://submit.ncbi.nlm.nih.gov/subs/sra/) with BioProject accession number (PRJNA1219831).

### Quantification of gene expression and differential expression analysis

Gene expression levels were estimated using RSEM (1.2.28 version) to map reads to the assembled transcriptome and counts obtained from these maps. Read counts were used for DESeq2 (1.26.0 version), to identify differentially expressed genes (DEGs) (Love et al. 2014). Resulting *P*-values were adjusted using the Benjamini and Hochberg’s approach for controlling false discovery rate. Differentially expressed genes had an absolute log_2_ FC ≥ 1 and an adjusted *P*-value ≤ 0.05. To identify DEGs under drought four comparisons were made: diploids versus tetraploids in control conditions (2x_C vs. 4x_C), diploids versus tetraploids under drought (2x_T vs. 4x_T), diploids under drought versus diploids in control conditions (2x_T vs. 2x_C), and tetraploids under drought versus tetraploids in control conditions (4x_T vs. 4x_C) (Fig. 3).

### Validation of candidate DEGs using RT-qPCR analysis

To validate RNA-seq results, 14 DEGs of the 2x_T versus 4x_T group were analyzed with qRT-PCR (Supplementary Table S2). RNA (2.0 µg) was reverse transcribed using Maxima™ H Minus cDNA Synthesis Master Mix with dsDNase (Thermo Fischer Scientific). qRT-PCR was carried out with PowerUp SYBR Green Master mix (Thermo Fischer Scientific), using primers listed in Supplementary Table S2. The validation subset was normalized with the 18S rRNA reference gene, and fold change was calculated by the 2^−∆∆CT^ method (Livak and Schmittgen 2001). The 2^−∆∆CT^ levels in stressed and non-stressed samples of single genotypes were estimated and correlated with the corresponding log_2_ FC obtained from RNA-seq.

### Gene ontology, KEGG enrichment and iTAK analysis

We conducted Gene Ontology (GO) functional enrichment analysis of DEGs with ShinyGO V0.80 (Ge et al. 2018). All DEGs were submitted to KOBAS (version 3.0, corrected *P*-value ≤ 0.05) to identify significantly enriched pathways in the KEGG database (Bu et al. 2021), choosing *M. truncatula* as the reference species. Gene functional enrichment was conducted using MapMan 3.6.0RC1 (https://mapman.gabipd.org/) (Thimm et al. 2004). DEGs were mapped using Mercator4 (7.0 version) (Lohse et al. 2014) to classify and predict functions and resulting maps analyzed with MapMan. Significant DEGs (padj ≤ 0.05) and their respective log_2_ fold change values were used for alignment with the Mercator map. iTAK was used to identify the transcription factor (TF) families among DEGs (Zheng et al. 2016). An adjusted *P*-value cutoff of 0.05 and an absolute Log_2_FC threshold of 1 were used to filter the significantly up- and down-regulated genes.

### Building gene co-expression networks

Co-expression network analysis identified gene clusters (modules) with highly correlated expression profiles (hub genes) using the Weighted Correlation Network analysis (WGCNA) package in R (Langfelder and Horvath 2008). The read counts matrix was filtered by retaining genes with more than 20 alignments in at least 3 samples. Data were normalized using DESeq2 median of ratios method (Love et al. 2014), setting ploidy and treatment as linear model variables. Scale-free topological analysis using the *pickSoftThreshold* function of WGCNA chose the proper soft-thresholding power (Langfelder and Horvath 2008). We selected the value closest to 0.9 to ensure a scale-free co-expression network when the soft threshold ranged from 1 to 20. A weighted adjacency matrix was constructed on the normalized data using automatic module detection function *blockwiseModules* of WGCNA (Langfelder and Horvath 2008) with the following parameters: net_type = signed, minModuleSize = 30, mergeCutHeight = 0.25, deepSplit = 2, corType = Pearson, randomSeed = 42, power = 28. Identified modules were corrected using a *k*-means clustering analysis with the *applyKMeans* function of *CoExpNets* (Botía et al. 2017). We used module eigengenes and gene significance to retrieve modules associated with specific ploidy-dependent experimental conditions.

SWItch Miner (SWIM) integrated network analysis was used to further screen significant hub genes (Paci et al. 2017) with an unweighted correlation network to identify master regulators associated with changes in the transcriptome. SWIM-based correlation network analysis was applied to predict genes affected by ploidy. An adjusted *P*-value cutoff of 0.05 and a log_2_fold change (log_2_FC) threshold of ± 1 were used to search for significant nodes based on Pearson correlation.

### Statistical analysis

Statistical analysis was performed using R-studio software (4.2.2 version). Two models, one linear and one linear mixed effect, were constructed. The sources of variation used in these models were: Genotype, Ploidy, Treatment, and Block. The term Genotype was nested within Ploidy, so different models were used to determine effects due to genotype or ploidy. Models included relevant interaction term Ploidy/Genotype x Treatment as a fixed effect. Block x Treatment was used as a fixed effect in linear models for validation and a random effect in linear mixed models for data analysis. All data and models were tested for normality and outlying data were removed as needed. Box plots were constructed to visualize the data. The measurements from the 3 leaves (technical replicates) were averaged to form one biological replicate (plant). The collected data were first processed using the randomized models for ANOVA analysis using R Studio. Pairwise analysis using EEMEANS was used to further explore interactions between ploidy and genotypes.

## Results

### Effect of drought and WGD on physiological and biochemical parameters

**Fig. 2:**
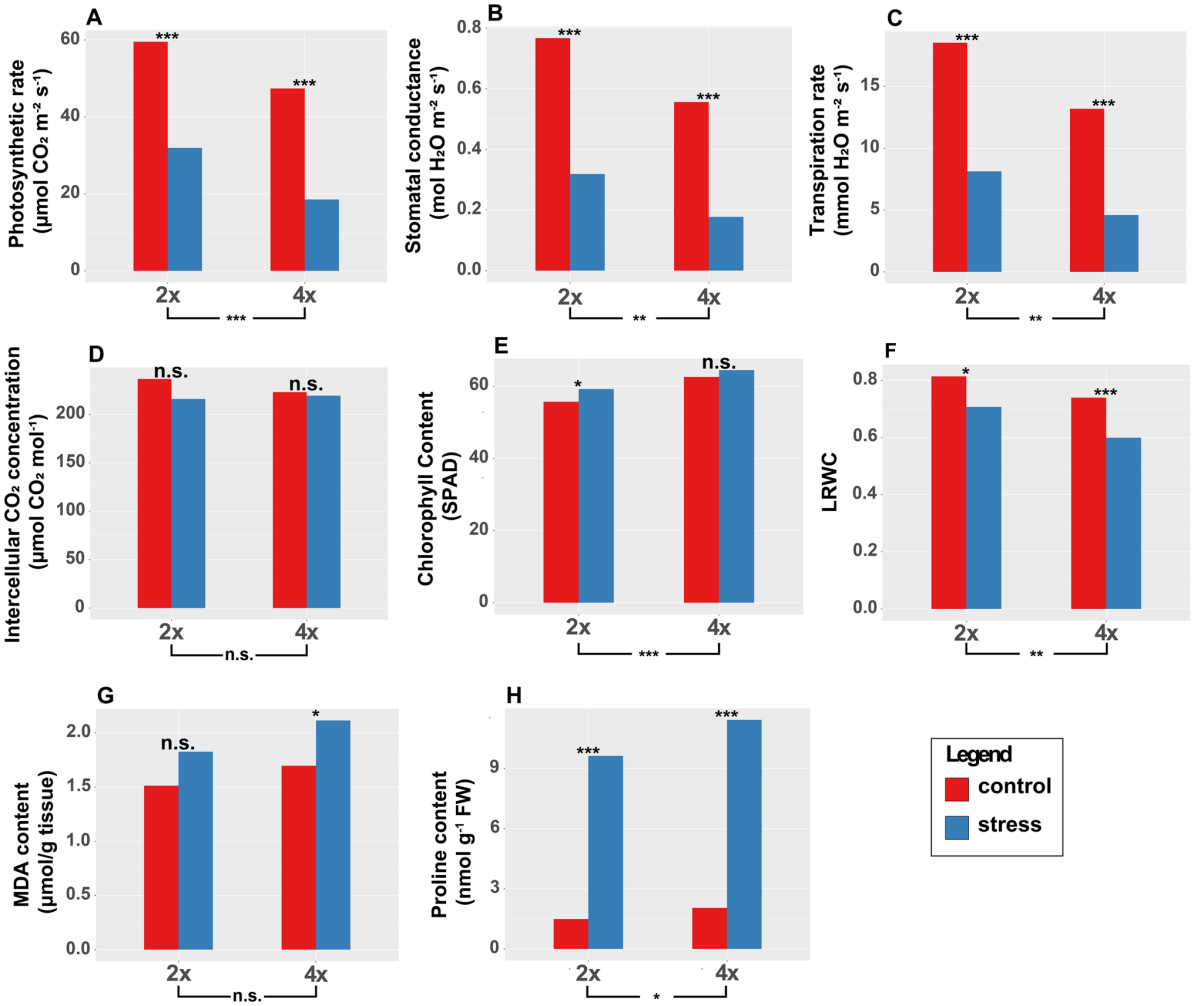
Physiological traits of 2x and 4x plants under control and drought conditions. *, ***, difference between stress and control significant at the *P* ≤ 0.05 or 0.001 level, according to the *F* test; n.s., not significant. The significance of differences between 2x and 4x plants is reported below each bar chart. No instances of significant Ploidy x Stress interactions were found. LRWC, leaf relative water content; MDA, malondialdehyde

Overall, drought significantly affected most physiological parameters (Fig. 2): lower photosynthetic rate, stomatal conductance, and transpiration were observed in both 2x and 4x plants. A strong increase in proline concentration was measured, while malondialdehyde (MDA) concentration was marginally increased in 4x plants only (Supplementary Table S3A, B; Supplementary Figs. S1–S8). Chlorophyll content was marginally higher in stressed plants, although not significantly (Fig. 2, Supplementary Fig. S1–S8).

Ploidy did statistically impact some physiological traits under both control and stress conditions (Fig. 2; Supplementary Table S3B, Supplementary Fig. S1–S8): photosynthetic rate, stomatal conductance, transpiration rate, and leaf relative water content were lower in 4x compared to 2x plants, whereas proline concentration and chlorophyll content (SPAD) were higher in 4x plants (Supplementary Table S3A, B). No significance was observed in the Stress × Ploidy interaction for any trait.

### Transcriptome assembly

After RNA-Seq and clean-up, a total of 850 million clean reads (Supplementary Table S1), representing 98.72% of the total reads, were obtained. Downstream analysis on roughly 23 million reads (7.09 Gb per sample), with high proportions of Q30 (95.03%) and GC content (42.07%) (Supplementary Table S1) and a relatively high *K*-mer coverage for de novo transcriptome assembly (min-kmer_cov = 5) yielded 246,789 transcripts with an N50 of 1944 bp and 91,141 unigenes with N50 of 1721 bp (Supplementary Table S1, Fig. S9A). Filtered unique reads were mapped to the reconstructed transcriptome with an average read mapping rate of 71.40%. The quality and completeness of our transcriptome assessed using the BUSCO tool (3.0.2 version) was comparable or higher than those reported for a de novo assembly (Simão et al. 2015; Wong et al. 2025). Among the 1440 searched BUSCO groups, in the whole transcriptome dataset, 87.5% complete transcripts, (27.78% single and 59.72% duplicated) were obtained (Figure S9B). In the non-redundant transcripts and the unigenes datasets, BUSCO values were 65.63% complete (63.99% single, 2.64% duplicated). Functional annotation of the unigenes with BLAST searches against public databases resulted in a total of 84,563 unigenes (92.78%) annotated in at least one database (Fig. S10).

### Identification of differentially expressed genes (DEGs)

**Fig. 3:**
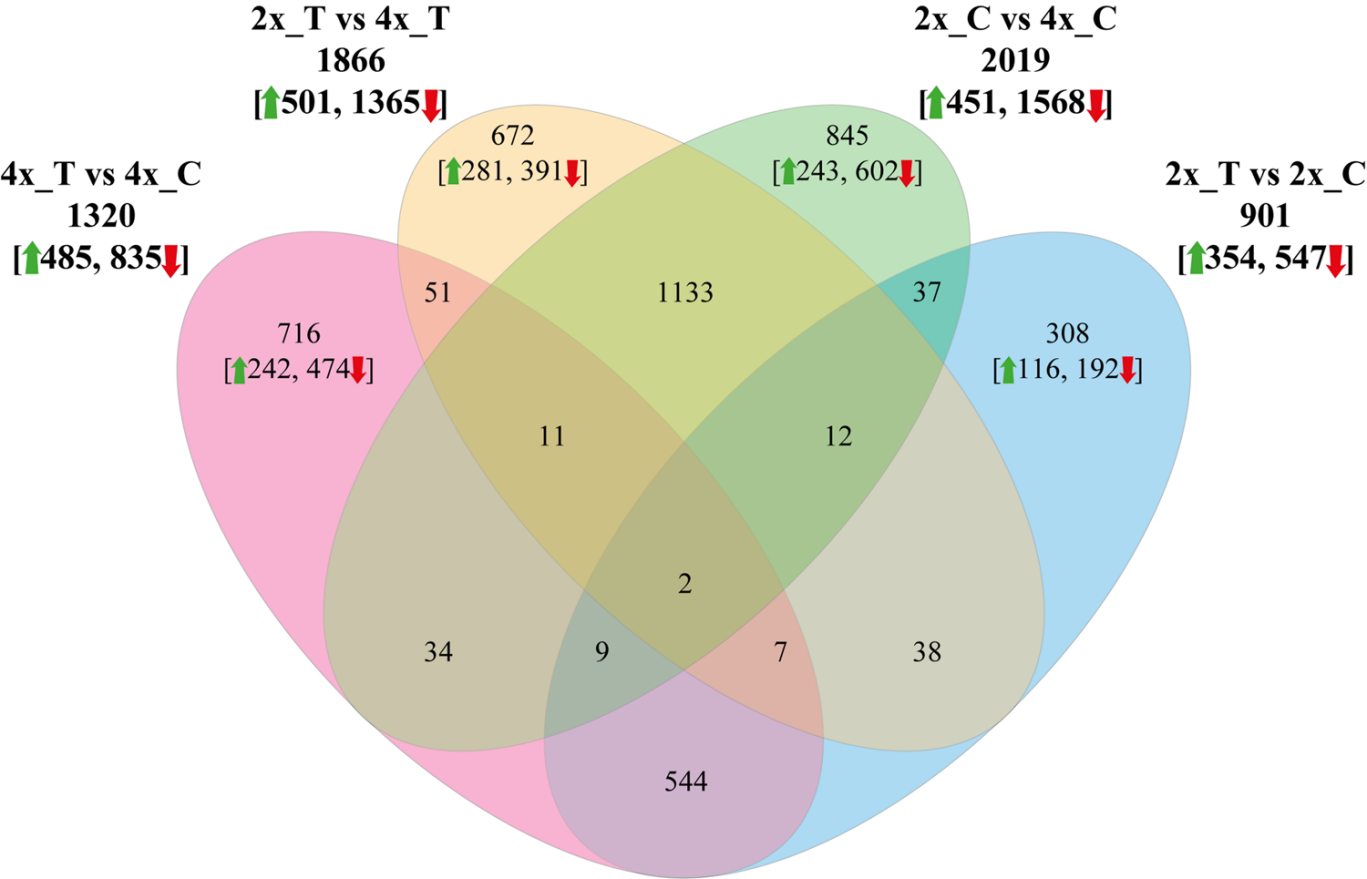
Venn diagram of significantly enriched DEGs under drought. Upward green arrows indicate significantly up-regulated genes, while downward red arrows indicate significantly down-regulated genes. Here, up-regulation corresponds to higher expression in the 2x compared to the 4x plants, that is, lower expression in the 4x plants

The number of genes whose expression levels changed between the experimental treatments are reported in Fig. 3. A majority of DEGs were downregulated by stress in both 4x plants (835/1320 = 64%) and 2x plants (547/901 = 63%), but the number of drought-affected genes was 46% higher in 4x plants (1320 vs. 901; Fig. 3; Supplementary Table S3). Considering the 2x_T versus 4x_T comparison, we found that 73% of DEGs (1365/1866) were upregulated in 4x with respect to 2x plants, the same percentage that exhibited upregulation in 4x versus 2x plants in control conditions (2x_C vs. 4x_C comparison: 1568/2019 = 73%). Therefore, the polyploid condition mostly brought about transcriptional upregulation irrespective of stress.

A total of 308 genes (116 up- and 192 down-regulated) were unique to the 2x_T versus 2x_C group (drought-sensitive in 2x plants, blue circle in Fig. 3), while 716 genes (242 up- and 474 down-regulated) were unique to the 4x_T versus 4x_C group (drought-sensitive in 4x plants, pink circle), indicating that polyploids responded to drought stress with a larger transcriptional modulation. We considered these 716 DEGs as candidates for being associated with ploidy-specific responses to drought.

The validation of expression levels for 14 selected DEGs was carried out by qRT-PCR. The results showed high congruence between RNA-Seq and qRT-PCR data (correlation coefficient r = 0.91, Fig. S11), confirming the high reliability of RNA-Seq.

### Functional analysis of DEGs involved in drought response

Ploidy-specific transcriptional differences in response to drought were further investigated through an enrichment analysis of Gene Ontology (GO) terms. Drought had different transcriptional consequences at 2x and 4x ploidies.

At the 4x level, the enriched GO terms unique to the group of 716 drought-affected DEGs (from the 4x_T vs. 4x_C comparison) were overwhelmingly related to plastid, organelle envelope, photosynthesis, water status, and stomata (24 of the 45 GO terms derived from more than 10 genes each, Supplementary Table S4). The terms “stomatal movement” (GO:0010118) and “regulation of stomatal movement” (GO:0010119) were unique to this group. The DEGs for these terms were mostly downregulated, with the exception of the term “response to water/water deprivation”, that derives from 13 up- and 4 down-regulated genes. Other highly represented, unique GO terms in the 4x_T versus 4x_C group were related to hormone/auxin (GO:0009737, GO:0009733, GO:0010817, GO:0042445). Terms related to proline (GO:0006560, GO:0006562 and GO:0010133), likely involved in osmotic stress response, were also unique to this group. Finally, two terms related to root development (GO:0048364, GO:0022622) and one with unidimensional cell growth (GO:0009826) were present.

A the 2x level, among the most represented GO terms specifically associated with drought response (group 2x_T vs. 2x_C) we found cation binding (GO:0043169, GO:0046872); regulation of gene expression/transcription/RNA (GO:0010468, GO:0051252, GO:0019219, GO:0006351, GO:0097659, GO:0032774), and the development of reproductive structures (GO:0048608, GO:0061458). Three terms related to hypoxia were also unique to this group (GO:0001666, GO:0036293, GO:0070482) (Supplementary Table S4).

**Fig. 4:**
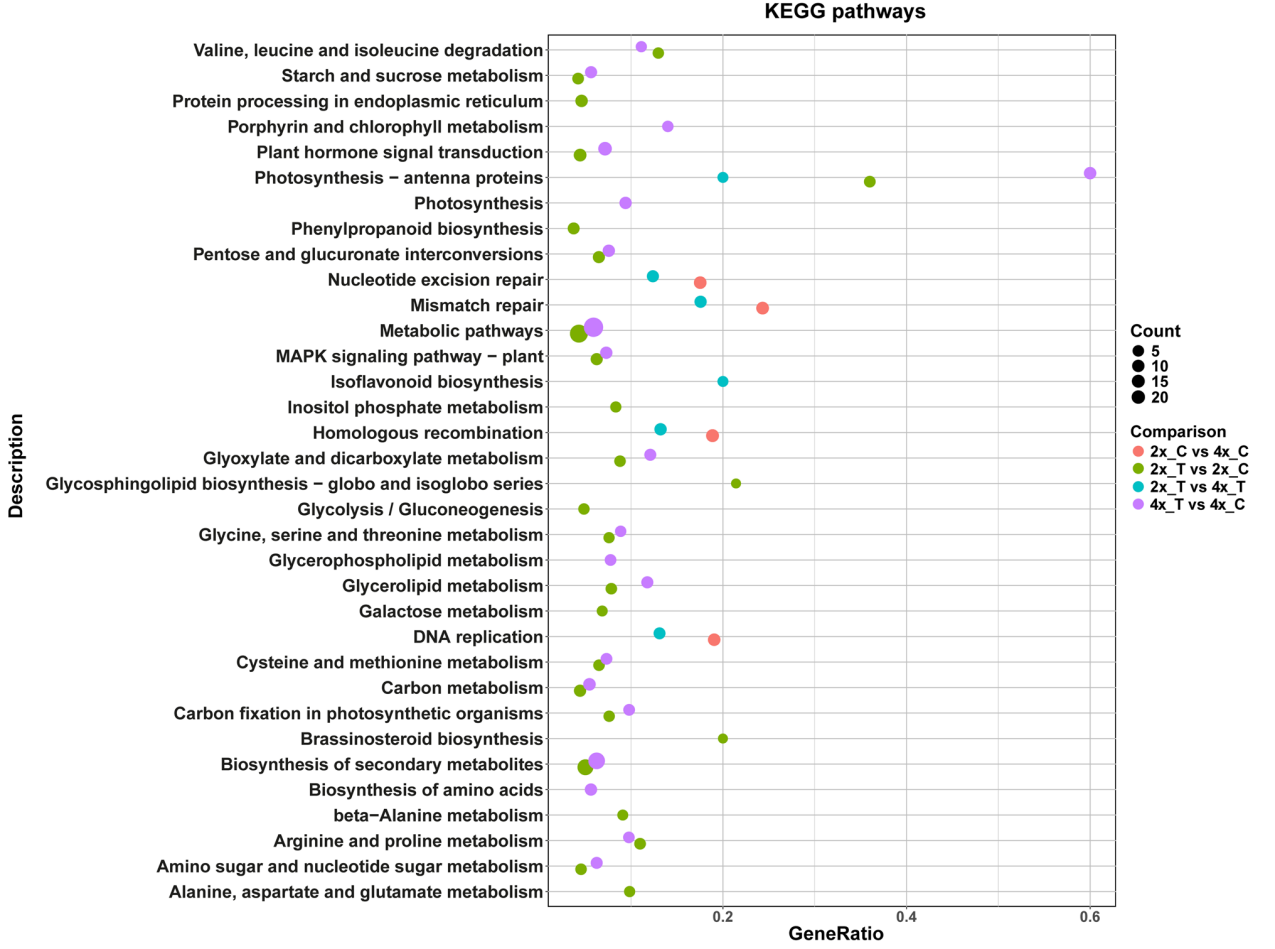
Kyoto Encyclopedia of Genes and Genomes (KEGG) enrichment analysis for the DEGs in the 2x_C versus 4x_C, 2x_T versus 4x_T, 2x_T versus 2x_C and 4x_T versus 4x_C groups. The enriched KEGG categories are listed, and the proportion of genes from the input dataset associated with each pathway relative to the total number of input genes are reported on the *X*-axis (Gene Ratio)

The DEGs from all four comparisons were mapped onto the KEGG database to detect WGD-responsive metabolic pathways. Figure 4 shows the main KEGG pathways sorted by decreasing significance value (adjusted *P*-value). Among the KEGG terms, “Metabolic pathways”, “Biosynthesis of secondary metabolites” and “Plant hormone signal transduction” represented the largest groups, which were specifically observed in stressed plants (Fig. 4).

While some pathways were enriched by drought at both ploidy levels (paired green and pink dots in Fig. 4), several pathways were enriched in a ploidy-specific fashion. In 2x plants, the pathways “Protein processing in endoplasmic reticulum”, “Phenylpropanoid biosynthesis”, “Inositol phosphate metabolism”, “Glycolysis/Gluconeogenesis” were enriched (isolated green dots in Fig. 4). In contrast, the “Porphyrin and chlorophyll metabolism and “Photosynthesis” pathways were only detected in 4x plants (isolated pink dots in Fig. 4). The “Photosynthesis—antenna proteins” pathway was detected in all stress-related groups of genes but presented a much larger gene ratio in the 4x_T versus 4x_C group (0.6, 15 DEGs) than in the 2x_T versus 2x_C group (0.36, 9 DEGs). These observations confirm the results of GO enrichment analysis (Supplementary Table S4), showing that photosynthesis and plastid functions were specifically involved in drought response of 4x plants. The pathways related to DNA functions (nucleotide excision repair, mismatch repair, homologous recombination, DNA replication) were only detected when 2x and 4x plants were compared, both in control and stress conditions (orange and blue dots are almost always associated in Fig. 4). The majority of the genes in these pathways were upregulated in 4x with respect to 2x plants.

Mapman analyses revealed 28 downregulated and 1 upregulated gene belonging to the “Photosynthesis–Photophosphorilation” pathways, which were uniquely found in the 4x_T versus 4x_C group. Several hormone-responsive genes involved in regulation and signal transduction were also overrepresented in this group (Supplementary Table S6).

In contrast, the most represented pathways in the 2x_T versus 2x_C group were related to chaperone activity and the proteasome (11 genes, the majority of which downregulated), both categorized under ‘protein homeostasis’. The second most numerous pathway in this group was RNA biosynthesis-transcriptional regulation (6 genes), with different types of transcription factors (see below) (Supplementary Table S6). Focusing on the drought response differences between ploidy levels (2x_T vs. 4x_T group), the two most represented Mapman pathways were Cell division (cell cycle organization, meiotic recombination and DNA replication, 6 genes, and Chromatin organization, 3 genes) and RNA (transcriptional regulation, 8 genes, and RNA homeostasis, 2 genes).

### Identification of transcription factor families responsive to drought

**Fig. 5:**
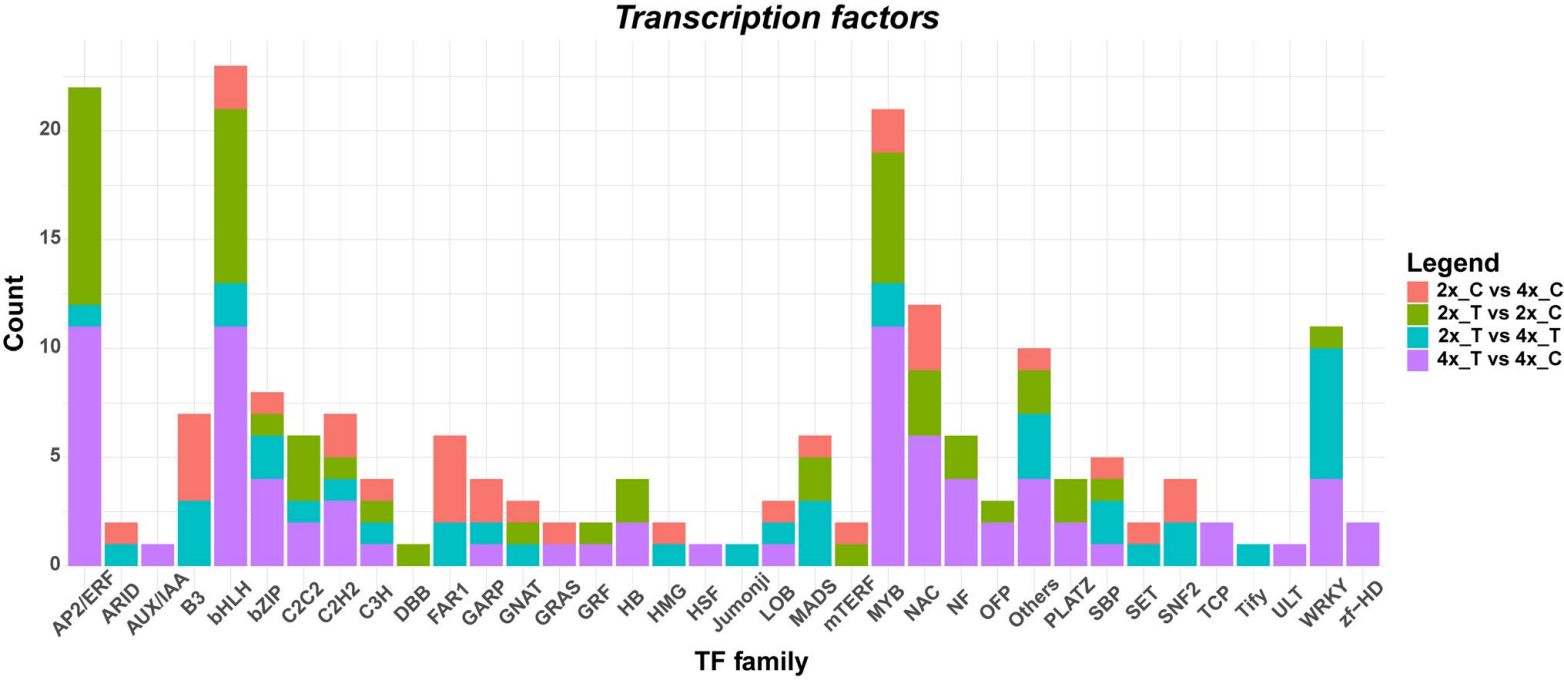
Distribution of transcription factors responsive to drought and/or WGD in the four groups of genes

We assessed the effect of WGD on transcription factor (TF) expression by examining the numbers of TFs present within the four groups of genes defined above. Seventy-nine TFs were modulated (59.5% downregulated, 40.5% upregulated) by the drought treatment in tetraploids (4x_T vs. 4x_C, purple bars in Fig. 5), more than in the other three groups of genes (33–50).

Among the TFs involved in drought response at both ploidy levels (2x_T vs. 2x_C and 4x_T vs. 4x_C groups, green and purple bars in Fig. 5), the most represented families were AP2/ERF, bHLH, MYB, NAC, and WRKY, known to play a pivotal role in drought responses (Joshi et al. 2016) (Fig. 5, Supplementary Table S7). By subtracting the DEGs belonging to the 2x_C versus 4x_C group from the 2x_T versus 4x_T group, we selected the DEGs that were specifically regulated by drought in neotetraploids. With this approach, we identified 23 TFs, belonging to 13 different families, with the WRKY family most represented (6 genes). Many of these TFs were downregulated in response to drought (Supplementary Table S7), corroborating the hypothesis that tetraploids may be more responsive to drought than diploids.

### Focusing on enriched GO terms for drought- and WGD-related genes

The analysis of the 2x_T versus 4x_T DEGs (Supplementary Table 8A–C) revealed 4x-upregulation of several genes associated with cell signalling, growth and development. These included *M. sativa* 6-phosphofructokinase (ortholog of *M. truncatula* PFK), *M. sativa* probable leucine-rich repeat receptor-like protein kinase At5g63930 isoform X2 (ortholog of *M. truncatula* LRR-RLK), *M. sativa* mitogen-activated protein kinase kinase kinase 20 (ortholog of *M. truncatula* MAPKKK20), and *M. sativa* wall-associated receptor kinase 2 (ortholog of *M. truncatula* WAK2). Transcripts involved in stress response were also upregulated in 4x plants: *M. sativa* heat shock cognate 70 kDa protein (ortholog of *M. truncatula* HSP70), *M. sativa* cold-regulated 413 inner membrane protein 1 (ortholog of *M. truncatula* COR413IM1) and *M. sativa* DMR6-LIKE OXYGENASE 1 isoform X1 (ortholog of *M. truncatula* DL01) (Supplementary file S8 A).

The analysis of the 4x_T versus 4x_C group DEGs revealed upregulation of *M. sativa* NAC domain-containing protein 55 (NAC055) transcription factor gene (ortholog of *A. thaliana* NAC055) (Supplementary Table S8C), previously shown to enhance drought tolerance in transgenic *Arabidopsis* (Mao et al. 2016). Transcription factors associated with water and osmotic stress were also upregulated, including *M. sativa* Ethylene-responsive transcription factor ERF053 (ortholog of *M. truncatula* ERF053), *M. sativa* Ethylene-responsive transcription factor RAP2-1 (ortholog of *A. thaliana* RAP2-1), *M. sativa* nuclear transcription factor Y subunit B-3 (ortholog of *M. truncatula* NFYB3) and *M. sativa* WRKY transcription factor 23 (ortholog of *M. truncatula* WRKY23) (Nelson et al. 2007; Li et al. 2022; Singh et al. 2023). Genes encoding for *M. sativa* probable galactinol–sucrose galactosyltransferase 1 (ortholog of *M. truncatula* RFS1), *M. sativa* ATP-dependent zinc metalloprotease FTSH 6 (ortholog of *M. truncatula* FTSH6), *M. sativa* bidirectional sugar transporter SWEET12 (ortholog of *M. truncatula* SWEET12) and *M. sativa* EARLY-RESPONSIVE TO DEHYDRATION 7 (ortholog of *M. truncatula* ERD7) were upregulated, consistent with their role in osmolyte homeostasis and water deprivation (Taji et al. 2002; Li et al. 2021; Aubry et al. 2024).

ABA-mediated stress-responsive genes were upregulated in the 4x_T versus 4x_C group, including *M. sativa* HVA22-like protein (ortholog of *M. truncatula* HVA22-like protein), *M. sativa* NAC2 transcription factor, *M. sativa* homeobox-leucine zipper protein ATHB-7, *M. sativa* Phosphatidylinositol-4-phosphate 5-kinase (ortholog of *M. truncatula* PIP5K) and *M. sativa* bHLH122-like (ortholog of *M. truncatula* bHLH122-like) consistent with their established functions in ABA-dependent water deficit relief (Zareen et al. 2024). Conversely, some key components of ABA signalling, such as *M. sativa* PYL4 and PYR1 (orthologs of *M. truncatula* PYL4 and PYR1) abscisic acid receptor were downregulated.

Downregulation was observed for genes related to photosynthesis within the 4x_T versus 4x_C group, including *M. sativa* Ribulose bisphosphate carboxylase/oxygenase activase (ortholog of *A. thaliana* RCA), *M. sativa* photosystem I chlorophyll a/b-binding protein 5 (ortholog of *M. truncatula* LHCA5), chloroplastic, *M. sativa* photosystem I subunit O isoform X1 (ortholog of *M. truncatula* PSAO), *M. sativa* psbQ-like protein 3 (ortholog of *M. truncatula* PQL3), *M. sativa* photosystem II oxygen-evolving enhancer protein 3 (ortholog of *M. truncatula* PSBQ3) and *M. sativa* photosystem II reaction center PsbP family protein (ortholog of *M. truncatula* PSBP) (Supplementary Table S8C). Other downregulated genes in the 4x_T versus 4x_C group were *M. sativa* photosynthetic NDH subunit of subcomplex B (ortholog of *M. truncatula* PNSB), *M. sativa* plastocyanin-like domain protein (ortholog of *M. truncatula* PETE), *M. sativa* Protein CURVATURE THYLAKOID 1B (ortholog of *A. thaliana* CURT1B), *M. sativa* protein ACTIVITY OF BC1 COMPLEX KINASE 8 (ortholog of *M. truncatula* ABC1K8), *M. sativa* thylakoid uminal 17.9 kDa protein (ortholog of *M. truncatula* TL17) and *M. sativa* protein PLASTID TRANSCRIPTIONALLY ACTIVE 16 (ortholog of *M. truncatula* PTAC16). Chlorophyll catabolism was also implicated by the upregulation of *M. sativa* pheophorbide *a* oxygenase (PAO) (ortholog of *M. truncatula* PAO) (Supplementary file S8 C) (Das et al. 2018). Since these genes were not found in the 2x_T versus 2x_C comparison, we infer greater sensitivity of the photosynthetic apparatus under drought in 4x compared to 2x plants, consistent with the observed decline in net photosynthesis (Fig. 2; Supplementary Fig. S1).

### Co-expression network analysis

**Fig. 6:**
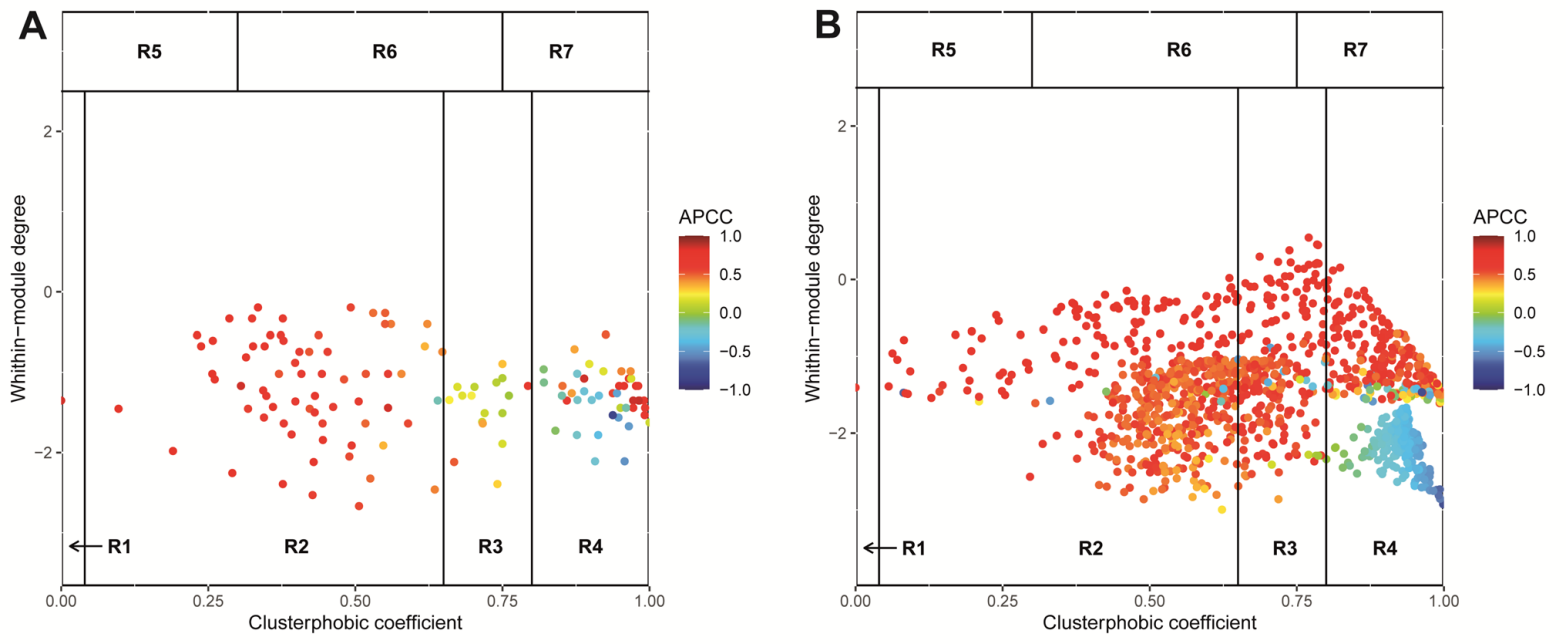
Heat cartography map of nodes generated by SWIM for the 2x_T versus 2x_C (**A**) and 4x_T versus 4x_C (**B**) datasets

SWIMer analysis was conducted to identify master regulators for drought response affected by genome doubling (Fig. 6A, B). In the 2x_T versus 2x_C group, 136 nodes were identified, 17 of them classified as switch genes (Supplementary Table S9; Fig. 6A). In contrast, as many as 232 switch genes were identified out of 1258 nodes in the 4x_T versus 4x_C group, confirming that drought induced greater transcriptomic perturbations in neotetraploid than diploid plants (Supplementary Table S9; Fig. 6B). Many switch genes identified in the 4x group are specifically related to the photosynthetic apparatus: several Chlorophyll a–b binding proteins, Ribulose bisphosphate carboxylase/oxygenase activase 2, the ribulose-1,5-bisphosphate carboxylase small subunit, and Photosystem II reaction center protein L (PsbL) (Supplementary Table S9).

The functional annotation of hub genes shared between 2x_T versus 2x_C and 4x_T versus 4x_C groups revealed GO terms associated with “response to stress” (GO:0006950), “response to abiotic stimulus” (GO:0009628), “regulation of hormone levels” (GO:0010817), “hormone metabolic process” (GO:0042445), and “cellular hormone metabolic process” (GO:0034754). We also identified stress-responsive categories such as “developmental process” (GO:0032502), “cell periphery” (GO:0071944), “anatomical structure development” (GO:0048856), and “plasma membrane” (GO:0005886) (Supplementary Table S9). Several processes related to drought response, including response to osmotic stress, transmembrane transport, photosynthetic apparatus regulation, and transcription factor activity, were specifically enriched in the 4x_T versus 4x_C group, confirming the results shown in Supplementary Table S4.

A Weighted gene co-expression network analysis (WGCNA R package) was conducted to identify modules that responded to drought differently at the two ploidy levels. We filtered genes with low expression levels (see above), resulting in retention of 34,310 genes across the entire dataset. These were grouped into 37 co-expression modules through *k*-means clustering analysis (Supplementary Fig. S12). In WGCNA, two key metrics were evaluated within each module: module membership (MM), which quantified the extent to which a gene’s expression pattern aligns with the module eigengene, and gene significance which reflected the degree of association between a gene and a specific condition (Langfelder and Horvath 2008).

**Fig. 7:**
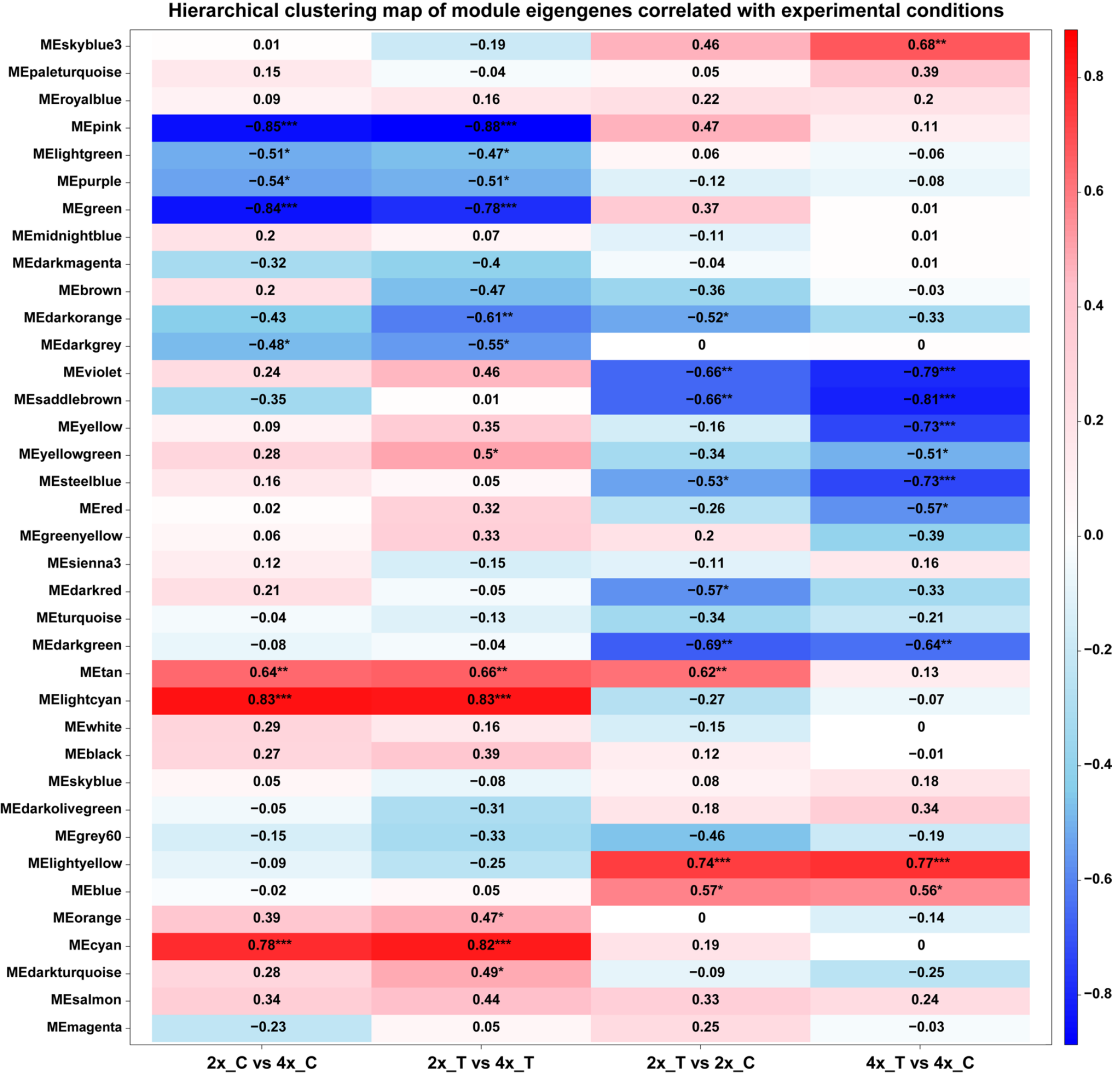
Correlation-Based Heatmap of Module Eigengenes (MEs) and the four groups of DEGs corresponding to the drought-WGD combinations by Pearson correlation. MEs represent the first principal component of the expression profiles of all genes within a module. Correlations between MEs and groups are calculated using a student’s *t*-distribution approach. Modules with a correlation *P*-value < 0.05 are marked with asterisks. The colour gradient indicates the strength and direction of the correlation: blue shades indicate negative correlations, while red or pink shades indicate positive correlations (colour figure online)

Correlation analysis across the 37 modules and the 4 comparisons (Fig. 7) revealed several modules showing consistent correlations with drought treatment (2x_T vs. 2x_C and 4x_T vs. 4x_C groups) either positive (lightyellow and blue) or negative (violet, seddlebrown, steelblue and darkgreen). Other modules showed correlations with WGD (2x_C vs. 4x_C and 2x_T vs. 4x_T groups), either positive (lightcyan, cyan) or negative (pink, green, lightgreen, purple, darkgrey). Overall, WGCNA did not identify modules significantly correlated with all four comparisons, indicating that WGD and drought drove largely independent transcriptional reprogramming.

Three modules (Skyblue3, yellow, yellowgreen and red) were specifically correlated with the 4x_T versus 4x_C comparison, while two modules (darkorange and darkred) were specifically correlated with the 2x_T versus 2x_C group (Fig. 7). We investigated the core gene sets of these six modules to identify specific or shared hubs. A gene encoding *M. sativa* late embryogenesis abundant protein 2 (ortholog of *M. truncatula* LEA2) was overexpressed in the yellowgreen module, consistent with its established roles in drought stress response (Magwanga et al. 2018). Two *M. sativa* Ribulose bisphosphate carboxylase/oxygenase activase 2 genes were observed in the yellow module and one *M. sativa* Photosystem II reaction center protein L (PsbL) (ortholog of *M. arabica* PsbL) gene was found in the yellowgreen module (Supplementary Table S10). These genes were also classified as switch genes by SWIM analysis (Supplementary Table S9), indicating their role in the neotetraploid photosynthetic network response to drought.

In the blue and lightyellow module, 5 genes encoding *M. sativa* delta-1-pyrroline-5-carboxylate synthetase (P5CS) (orthologs of *M. truncatula* P5CS), a crucial enzyme in the biosynthesis of proline (Kaur and Asthir 2015), were upregulated (Supplementary Table S10). A gene encoding for *M. sativa* delta-1-pyrroline-5-carboxylate dehydrogenase 12A1 (ortholog of *M. truncatula* ALDH12A1) was also upregulated. These observations align with the strong increase of proline concentration induced by drought (Fig. 2; Supplementary Fig. S8) (Yoshiba et al. 1997; Deuschle et al. 2001; Zhang et al. 2014). Several DNA-binding transcription factor genes belonging to the blue and lightyellow modules were overexpressed, including *M. sativa* nuclear transcription factor Y subunit B-3 (ortholog of *M. truncatula* NFYB3), *M. sativa* Ethylene-responsive transcription factors ERF034 and RAP2-1 (orthologs of *M. truncatula* ERF034 and RAP2-1), *M. sativa* squamosa promoter-binding protein 1 (ortholog of *M. truncatula* SPL1), *M. sativa* NAC domain-containing protein 104 (ortholog of *M. truncatula* NAC104), *M. sativa* NAC transcription factor 29 (ortholog of *A. thaliana* NAC29), *M. sativa* Dehydration-responsive element-binding protein 3 (ortholog of *A. thaliana* DREB3), *M. sativa* transcription factor bHLH122-like protein (ortholog of *M. truncatula* bHLH122-like protein) and *M. sativa* transcription factor MYB17 (ortholog of *M. truncatula* MYB17) (Supplementary Table S10).

Among the most significant genes in the darkorange and darkred modules (correlated with the 2x_T vs. 2x_C dataset), several receptor-like serine/threonine-protein kinases involved in defence responses were downregulated (Supplementary Table S10).

## Discussion

The impact of drought on global food security is increasingly evident (Chaffai et al. 2024), making the improvement of drought tolerance a priority in crop species, including alfalfa (Al-Khayri et al. 2016; Humphries et al. 2021). WGD has shown the potential to enhance stress tolerance through a complex interplay of genomic, physiological, and molecular mechanisms encompassing genome buffering, greater flexibility in gene expression, and epigenetic modifications (Doyle and Coate 2019; Heslop-Harrison et al. 2023). Therefore, this study on the effects of drought on 2x and 4x full sib alfalfa plants was undertaken to test the hypothesis of polyploid advantage and the immediate impacts of WGD.

The concept of conducting genetic studies and breeding programs at the 2x level and transferring the results to the 4x cultivated materials through 2n gametes was introduced many years ago (Chase 1963, 1964; McCoy and Bingham 1988). For example, in potato, there are several examples of sourcing genes conferring tolerance to abiotic stress for introgression into 4x cultivated potato by ploidy manipulation through unilateral sexual polyploidization (4x × 2x or 2x × 4x crosses) (Carputo et al. 2000).

Wild, diploid *M. sativa* can be a valuable source of stress tolerance genes (Humphreys et al. 2021) for introgression into 4x alfalfa cultivars via 2n gametes. However, ploidy level can affect gene expression (as shown in the alfalfa plants studied here, Santoro et al. 2025), and it cannot be assumed that the effect of drought tolerance genes (in the case of our study) are the same in both 2x and 4x plants. Therefore, studying the impact of drought stress on gene expression in full sib plants at two ploidy levels is a preliminary step to the establishment of breeding programs aimed at introgressing genes from 2x to 4x alfalfa. In the longer term, our results could help to identify drought tolerance genes that are either not impacted by WGD or are enhanced by WGD to use as breeding targets for improved adaptation to drought (Tossi et al. 2022).

In this experiment, we found that physiological and biochemical traits were significantly affected by drought in both 2x and 4x plants, but the differences between ploidies were small and nonsignificant, indicating that, in our experimental conditions, WGD alone did not improve nor reduce drought tolerance. Lower photosynthetic rate, stomatal conductance, transpiration and leaf relative water content per unit leaf area was observed in 4x compared to 2x under both drought and control conditions, accompanied by slightly higher chlorophyll content. In a previous work, we found that in the leaves of the studied plants, stomata density was about 30% lower in 4x plants relative to 2x plants, and stomata surface was about 30% larger (Rosellini et al. 2016). Thus, gas exchange and consequently photosynthesis per unit leaf area are deeply affected by WGD.

In control conditions, we observed that 4x plants had lower physiological parameters than 2x plants: photosynthetic rate (− 20.83%), stomatal conductance (− 27.51%), and transpiration rate *per unit leaf area* (− 28.83%). Since the total leaf surface is 35% larger in 4x plants (Rosellini et al. 2016), we calculated the physiological parameters *per leaf* in the 4x plants by multiplying all parameters by 1.35, obtaining slightly higher values in 4x compared to 2x plants. The overall picture that we can draw from gas exchange-based photosynthesis-related traits is that 4x plants had lower photosynthetic potential than 2x plants per unit leaf area, but their larger leaves allowed them to outperform the *per leaf* photosynthetic potential of 2x plants. Our data appear in contrast with older studies reporting that the *M. sativa* photosynthetic rate per unit leaf area did not change with WGD (Meyers et al. 1982a, b; Molin et al. 1982). However, studies in *Fragaria* (Gao et al. 2017) appear to confirm our findings, showing that wild 4x genotypes have lower net photosynthetic rates, transpiration rates and stomatal conductivity compared with 2x genotypes.

Interestingly, in 4x plants under drought stress we observed a larger, though not significantly, reduction of physiological parameters than in 2x plants: photosynthetic rate (− 41.96%), stomatal conductance (− 44.38%) and transpiration rate (− 43.43%). The concomitant downregulation of hundreds of genes involved in photosynthesis and stomatal movement suggests that WGD made the 4x plants more responsive to drought. This hypothesis is consistent with a recent study of water allocation across four ploidy levels in the *Dianthus broteri* species complex, indicating a shift in water use strategy with greater allocation to roots and xylem, with the potential to increase water uptake under water limiting conditions (Lòpez-Jurado et al. 2025). Regulating stomatal conductance to control CO_2_ uptake and water loss has been observed as a response to drought in *ssp falcata:* some wild *ssp. Falcata* populations had lower stomatal conductance (Ray et al. 2004) and higher leaf chlorophyll content under severe drought compared to domesticated alfalfa (Hanson 2015), indicating ability to delay leaf senescence and retain water under drought*.*

In this study, the neotetraploids showed a distinctive transcriptomic response to drought compared to diploids, possibly connected with controlling water loss from their larger cells and stomata, thus conserving water in the face of stress. The scope of this experiment does not allow us to make statements on long term impacts of WGD on drought response but reveals an immediate shift at the transcriptional level.

Further looking into the transcriptome through gene ontology and pathway enrichment analyses, we identified several hundred transcripts that were specifically associated with the drought response of neopolyploid plants. The most notable finding was the involvement of genes related to plastids and photosynthesis in the 4x response to drought, which were not found at the 2x level, indicating that WGD affected photosynthesis under stress (Warner and Edwards 1993; Coate et al. 2012).

Five genes encoding Δ-pyrroline-5-carboxylate synthetase (P5CS), a key enzyme of proline biosynthesis, were found to be upregulated in both 2x and 4x plants under drought, confirming that proline accumulation is a mechanism for overcoming osmotic stress under drought (Ozturk et al. 2021). However, at the biochemical level, we found higher proline levels in 4x than in 2x plants, both in control (+ 37.56%) and drought conditions (+ 18.65%). This indicates that the 4x plants require a higher osmolyte concentration creating a lower constitutive osmotic potential level, possibly due to 4x cells having a higher volume-to-surface ratio (Doyle and Coate 2019). In yeast, transgenic increase of proline content brought about polyploidy (Maggio et al. 2002), suggesting that a relationship may exist between proline concentration and cell volume. This may help to explain upregulation of a gene encoding Δ^−1^-pyrroline-5-carboxylate dehydrogenase (associated with protection from proline toxicity, Deuschle et al. 2001) that we observed in 4x plants exposed to drought. In summary, WGD per se may require an increase in proline concentration due to increased cell volume, and this may affect drought stress response.

Transcriptomic analyses also revealed that a set of phytohormone genes responded to drought in a ploidy-independent manner. The cytokinin receptor histidine kinase AHK3 was up-regulated at both ploidy levels: it is known to function as a negative regulator of ABA and osmotic stress signalling in a cytokinin-dependent manner (Tran et al. 2007, 2010). This suggests a regulatory mechanism where ABA-responsive pathways are activated while signalling components are modulated to fine-tune the stress response (Valdés et al. 2012; Dittrich et al. 2019). Additionally, both the F-box protein GID2-like and gibberellin receptor GID1b were found to be upregulated. These proteins are integral to the gibberellin signal transduction cascade, facilitating DELLA degradation and enhancing stress resilience by modulating growth and environmental response mechanisms (Tran and Pal 2014). Transcription factor families that are well-established mediators of drought response in plants AP2/ERF, bHLH, MYB, NAC, and WRKY (Joshi et al. 2016) were overrepresented among WGD-related DEGs under drought. We also found down-regulation of ABA biosynthetic genes. These findings suggest a regulatory network that balances ABA levels at 4x level in response to drought differently than in 2x plants (Deuschle et al. 2001; Kuromori et al. 2018; Shrestha et al. 2021). We also found that in neotetraploid plants under drought, genes implicated in cell wall signalling, growth and development were mostly downregulated. This suggests that in the larger cells of 4x plants, the cell walls need to comply with drought with transcriptional changes (Cosgrove 2005; Tenhaken 2015).

Lastly, it should be mentioned that comparison of 2x with 4x plants in control conditions revealed that more DEGs were up-regulated than down-regulated in 4x plants (73%). These data confirm previous findings in the same plant materials (Santoro et al. 2025) and indicates that neopolyploidization brings about a more than proportional transcription level change on a per-cell basis for about 2000 genes (2,019 DEGs in control conditions, 1,866 DEGs in drought conditions). Also, the number of genes that were specifically affected by WGD (845) was similar to what we observed for the same plants in different environmental conditions (714 ploidy-sensitive genes, Santoro et al. 2025).

## Conclusions

This study delved into the effects of WGD on alfalfa response to drought. While phenotypic traits, such as photosynthetic rate, stomatal conductance, and transpiration were similarly affected by drought in 2x and 4x plants, their transcriptomes diverged to a significant extent, indicating that WGD brings about a new transcriptional equilibrium for basic functions such as photosynthesis, osmotic balance, and hormonal signalling, but this did not translate into significant phenotypic divergence in response to drought. In other words, different transcriptional landscapes lead to similar phenotypes of 4x and 2x plants in the tested drought conditions. These results indicate that WGD does not immediately enhance tolerance to drought in alfalfa, though larger scale field studies would be necessary to confirm these findings. On the other hand, it cannot be excluded that WGD sets the stage for long-term adaptation to drought, given the transcriptional novelty that we observed. We are currently testing other abiotic stress types to gain a more comprehensive picture of the impact of WGD on stress tolerance in alfalfa.

## Supplementary Information

Below is the link to the electronic supplementary material.

Supplementary file1 (DOCX 15 KB)

Supplementary file2 (XLSX 13 KB)

Supplementary file3 (DOCX 22 KB)

Supplementary file4 (XLSX 2606 KB)

Supplementary file5 (XLSX 176 KB)

Supplementary file6 (XLSX 80 KB)

Supplementary file7 (XLSX 108 KB)

Supplementary file8 (XLSX 303 KB)

Supplementary file9 (XLSX 649 KB)

Supplementary file10 (XLSX 15723 KB)

Supplementary file11 (DOCX 743 KB)

Supplementary file12 (DOCX 335 KB)

Supplementary file13 (DOCX 193 KB)

Supplementary file14 (DOCX 66 KB)

Supplementary file15 (DOCX 113 KB)

## Data Availability

Transcriptome data for this project has been submitted to NCBI-SRA (https://submit.ncbi.nlm.nih.gov/subs/sra/) with BioProject accession number (PRJNA1219831). All other data is available upon request.
