# Supplementary material for: Whole genome duplication drives transcriptome reprogramming in response to drought in alfalfa
Source: Plant Cell Rep. 2025 Sep 9;44(10):209. doi: 10.1007/s00299-025-03593-9 (PMC12417302; doi:10.1007/s00299-025-03593-9)
Supplement: Supplementary file 1 — Supplementary file1 (DOCX 15 KB) [file 299_2025_3593_MOESM1_ESM.docx]

**Table S1.** Summary statistics of the RNA quality and sequencing results.

| Average RIN | 6.6 |
| --- | --- |
| Clean reads | 850 million |
| Number of transcripts | 246,789 |
| Number of unigenes | 91,141 |
| Average read-mapped rate | 71.40 % |
| Transcripts N50, bp | 1944 |
| Unigenes N50, bp | 1721 |
| Q30 % | 95.03 |
| QC content % | 42.07 |
