# Supplementary material for: Whole genome duplication drives transcriptome reprogramming in response to drought in alfalfa
Source: Plant Cell Rep. 2025 Sep 9;44(10):209. doi: 10.1007/s00299-025-03593-9 (PMC12417302; doi:10.1007/s00299-025-03593-9)
Supplement: Supplementary file 3 — Supplementary file3 (DOCX 22 KB) [file 299_2025_3593_MOESM3_ESM.docx]

**Table S3 A.** Summary of Anova results. Significance of the effects of Genotype, Treatment and Genotype:Treatment interaction (*, **, ***: P<0.05, 0.01 and 0.01, respectively)

| **Parameters** | **Main effect** | | **Interaction** |
| --- | --- | --- | --- |
|  | ***G*** | ***T*** | ***G x T*** |
| Photosynthetic rate | *** | *** | n.s. |
| Stomatal conductance | ** | ** | n.s. |
| Intercellular CO_2_ concentration | * | n.s. | n.s. |
| Transpiration rate | *** | ** | n.s. |
| Clorophyll content (SPAD) | *** | * | n.s. |
| Leaf relative water content | ** | *** | n.s. |
| Proline concentration | *** | *** | * |
| Malondialdehyde concentration | *** | ** | n.s. |

**Table S3 B.** Summary of Anova results. Significance of the effects of Ploidy, Treatment (water shortage) and Ploidy:Treatment interaction (*, **, ***: P<0.05, 0.01 and 0.01, respectively).

| **Parameters** | **Main effect** | | **Interaction** |
| --- | --- | --- | --- |
|  | ***P*** | ***T*** | ***P x T*** |
| Photosynthetic rate | *** | *** | n.s. |
| Stomatal conductance | ** | *** | n.s. |
| Intercellular CO_2_ concentration | n.s | n.s | n.s. |
| Transpiration rate | ** | ** | n.s. |
| Chlorophyll content (SPAD) | *** | * | n.s. |
| Leaf relative water content | ** | *** | n.s. |
| Proline concentration | * | *** | n.s. |
| Malondialdehyde concentration | n.s. | * | n.s. |

| **Table S3 C**: Averages of physiological and biochemical traits of diploid and tetraploid plants in control conditions. | | | | |
| --- | --- | --- | --- | --- |
| *,**, ***: significantly different at P<0.05, 0.01, 0.001 according to ANOVA | | | | |
|  | **Diploid** |  | **Tetraploid** | Significance |
| **Trait** |  |  |  |  |
| Photosynthetic rate^a^ | 59.517 |  | 47.386 | ** |
| Stomatal conductance^b^ | 0.767 |  | 0.556 | 0.074 |
| Transpiration rate^c^ | 18.539 |  | 13.194 | * |
| Intercellular CO2^d^ | 236.666 |  | 223.066 | 0.396 |
| Chlorophyll content^e^ | 55.702 |  | 62.588 | *** |
| LRW | 0.814 |  | 0.739 | * |
| MDA^f^ | 1.513 |  | 1.697 | 0.245 |
| Proline^g^ | 1.49 |  | 2.05 | 0.325 |
| Units- a: μmol CO_2_ mol^-1^, b: mol H_2_O m^-2^s^-2^, c: mmol H_2_O m^-2^s^-2^,  d: μmol CO_2_ mol^-1^, e: SPAD, f: μmol/g tissue, g; nmol g^-1^ FW | | | | |
|  |  |  |  |  |
|  |  |  |  |  |

| **Table S3 D**: Averages of physiological and biochemical traits of diploid and tetraploid plants in control conditions. | | | | |
| --- | --- | --- | --- | --- |
| *,**, ***: significantly different at P<0.05, 0.01, 0.001 according to ANOVA | | | | |
|  | **Diploid** |  | **Tetraploid** | Significance |
| **Trait** |  |  |  |  |
| Photosynthetic rate^a^ | 31.965 |  | 18.551 | *** |
| Stomatal conductance^b^ | 0.319 |  | 0.177 | *** |
| Transpiration rate^c^ | 8.134 |  | 4.601 | *** |
| Intercellular CO2^d^ | 216.022 |  | 219.4 | 0.828 |
| Chlorophyll content^e^ | 59.23 |  | 64.468 | ** |
| LRW | 0.707 |  | 0.599 | * |
| MDA^f^ | 1.826 |  | 2.114 | 0.899 |
| Proline^g^ | 9.63 |  | 11.4 | 0.332 |
| Units- a: μmol CO_2_ mol^-1^, b: mol H_2_O m^-2^s^-2^, c: mmol H_2_O m^-2^s^-2^,  d: μmol CO_2_ mol^-1^, e: SPAD, f: μmol/g tissue, g; nmol g^-1^ FW | | | | |
|  |  |  |  |  |
|  |  |  |  |  |
