# Supplementary material for: Whole genome duplication drives transcriptome reprogramming in response to drought in alfalfa
Source: Plant Cell Rep. 2025 Sep 9;44(10):209. doi: 10.1007/s00299-025-03593-9 (PMC12417302; doi:10.1007/s00299-025-03593-9)
Supplement: Supplementary file 11 — Supplementary file11 (DOCX 743 KB) [file 299_2025_3593_MOESM11_ESM.docx]

**Whole genome duplication drives transcriptome reprogramming in response to drought in alfalfa**

Plant Cell Reports

Santoro D.F., Anderson A. W., Alavi S.N., Malatesta Pierleoni V.A., Rosellini D.*

*corresponding author: [daniele.rosellini@unipg.it](mailto:daniele.rosellini@unipg.it)

Department of Agricultural, Food and Environmental Sciences - University of Perugia, Borgo XX giugno 74, 06121 Perugia, Italy.

Interuniversity Consortium for Biotechnology (CIB), Area Science Park, Padriciano 99, 34149 Trieste, Italy

**Supplementary figures S1-S8**. Physiological parameters of 2x and 4 alfalfa plants

**
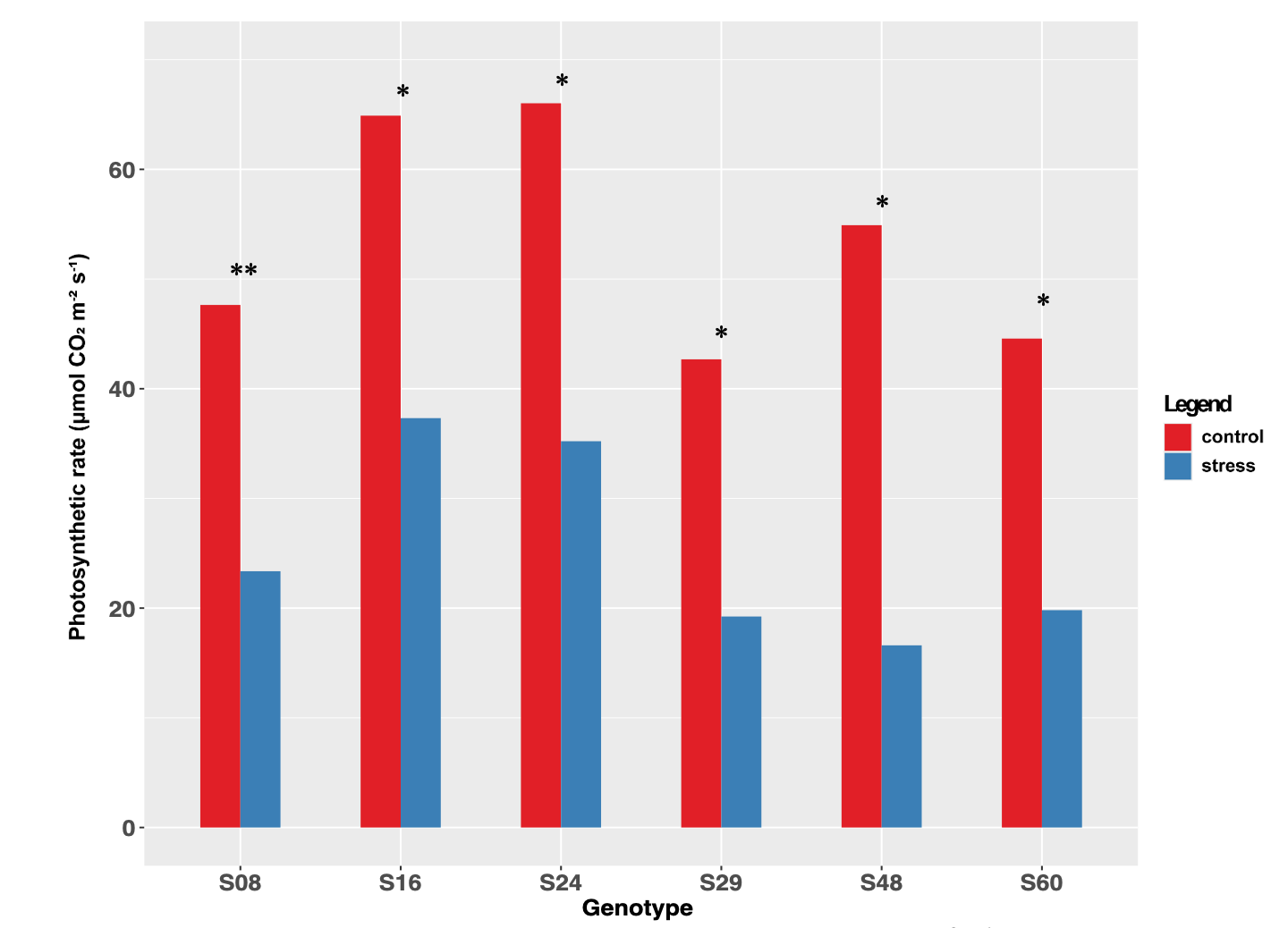
Figure S1.** Effect of water stress on the photosynthetic rate (µmol CO_2_ m^-2^ s^-1^).


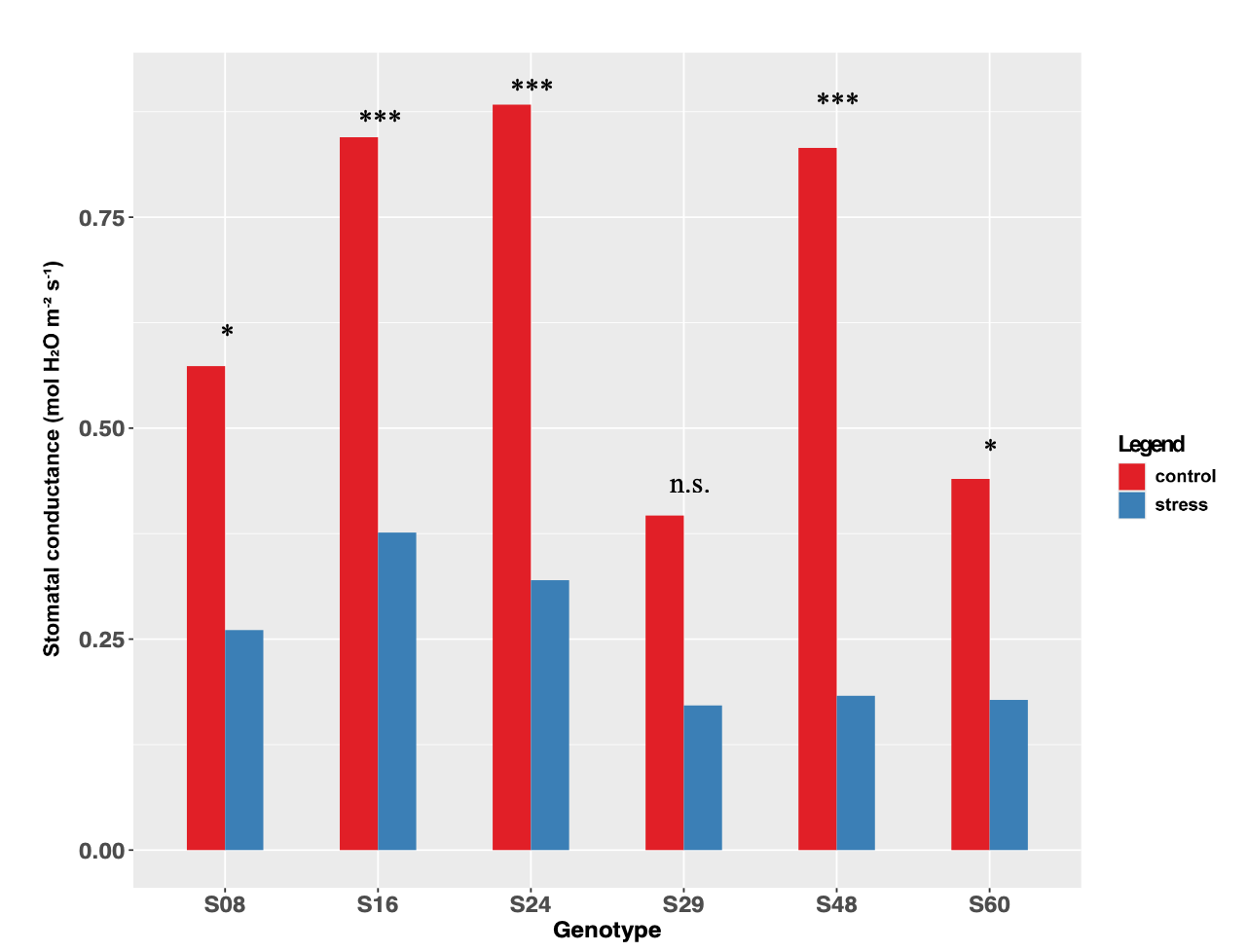


**Figure S2.** Effect of water stress on stomatal conductance (conductance (mol H_2_O m^-2^ s^-1^).

**
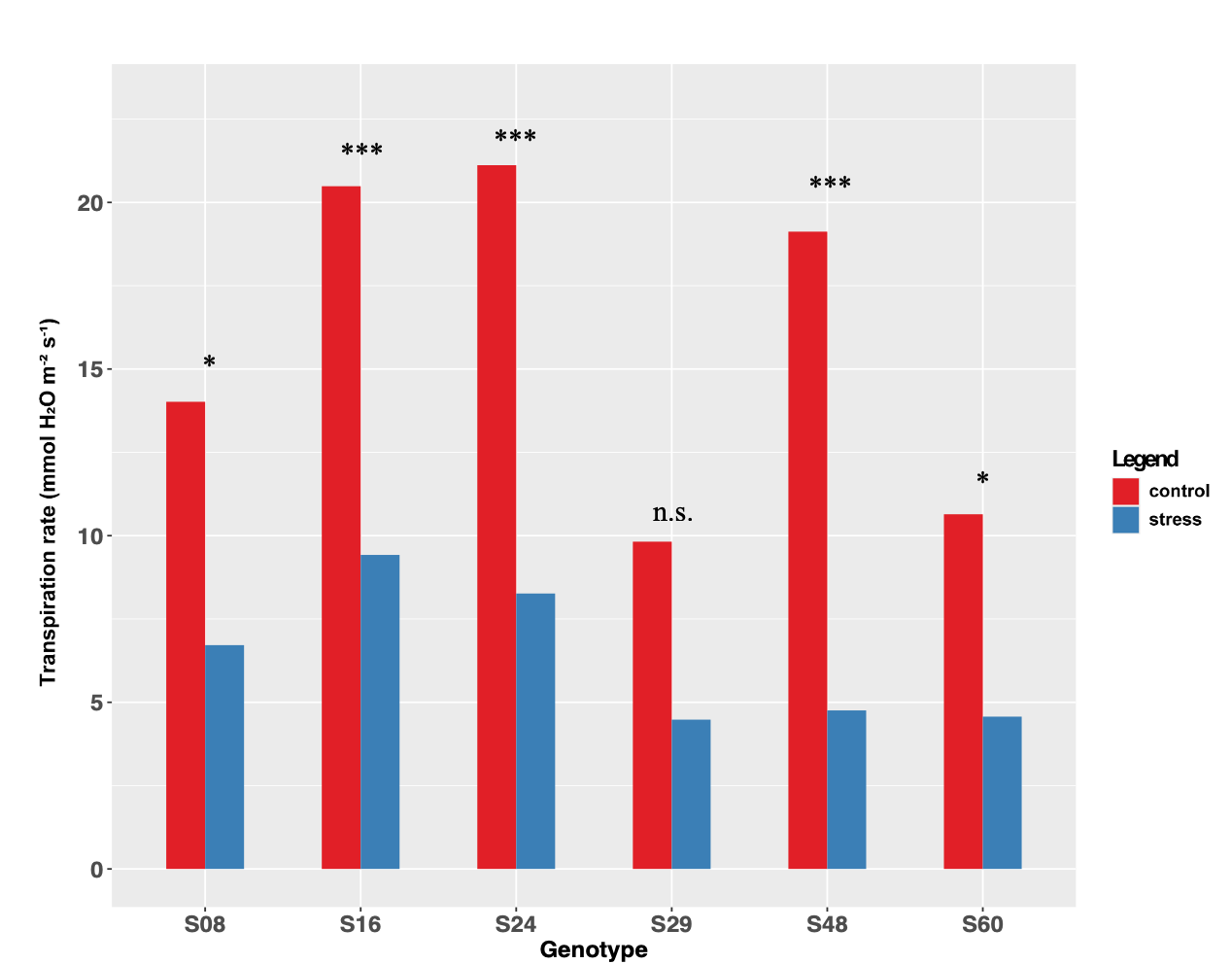
Figure S3.** Effect of water stress on the transpiration rate (mmol H_2_O m^-2^ s^-1^).


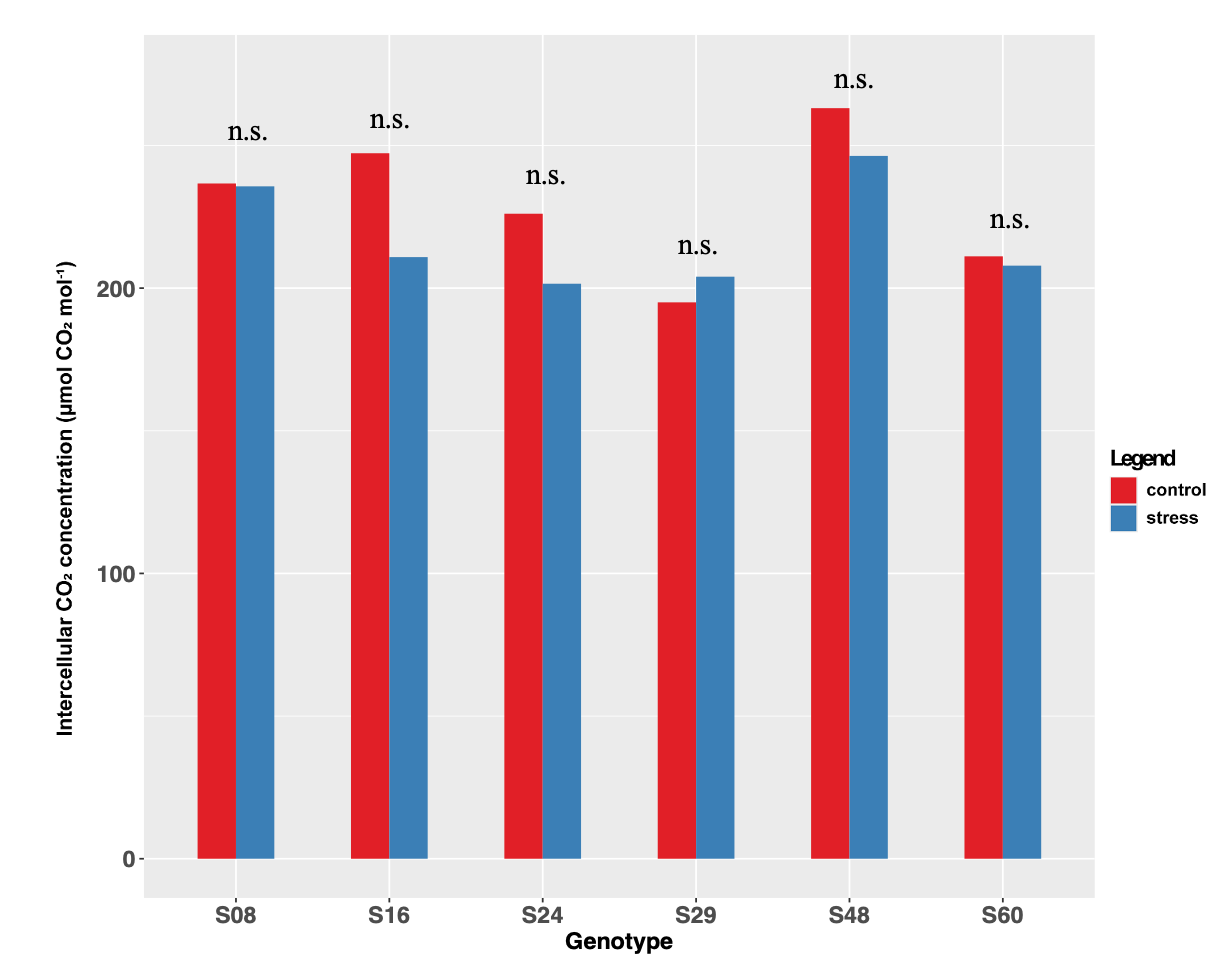


**Figure S4.** Effect of water stress on the intracellular CO_2_ concentration (µmol CO_2_ mol^-1^).


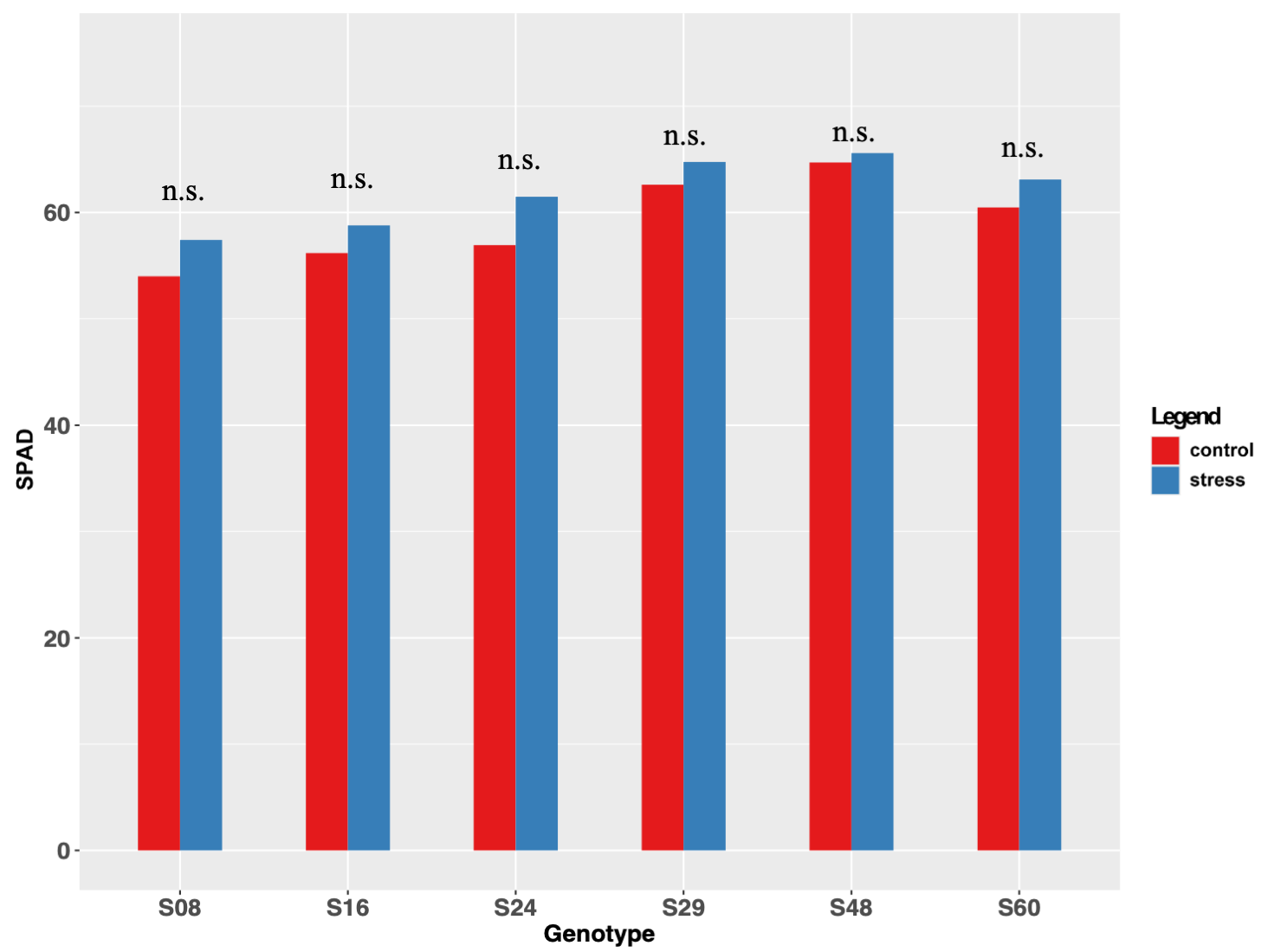


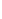


**Figure S5.** Effect of water stress on chlorophyll content (SPAD).

**
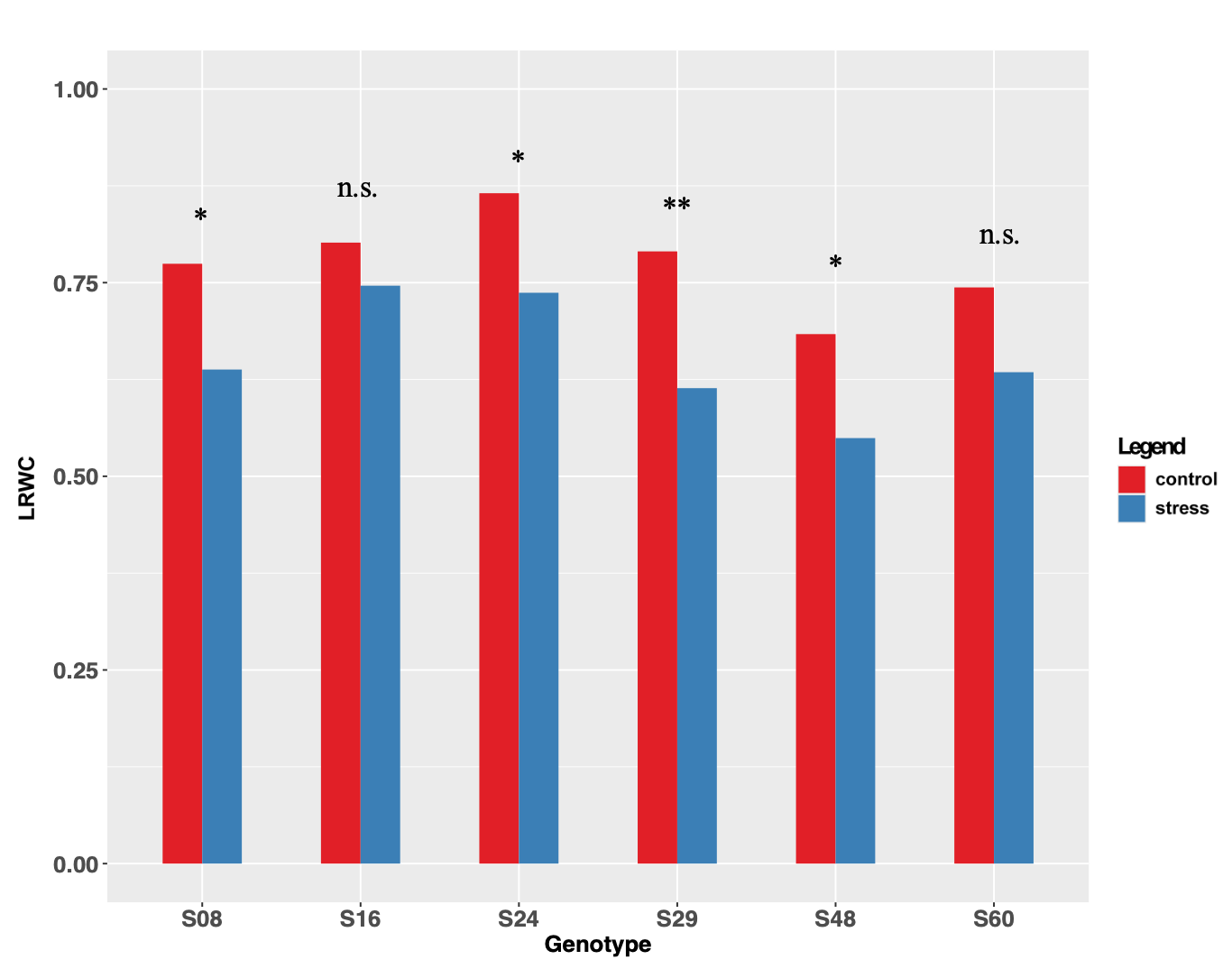
Figure S6.** Effect of water stress on the leaf relative water content.

**
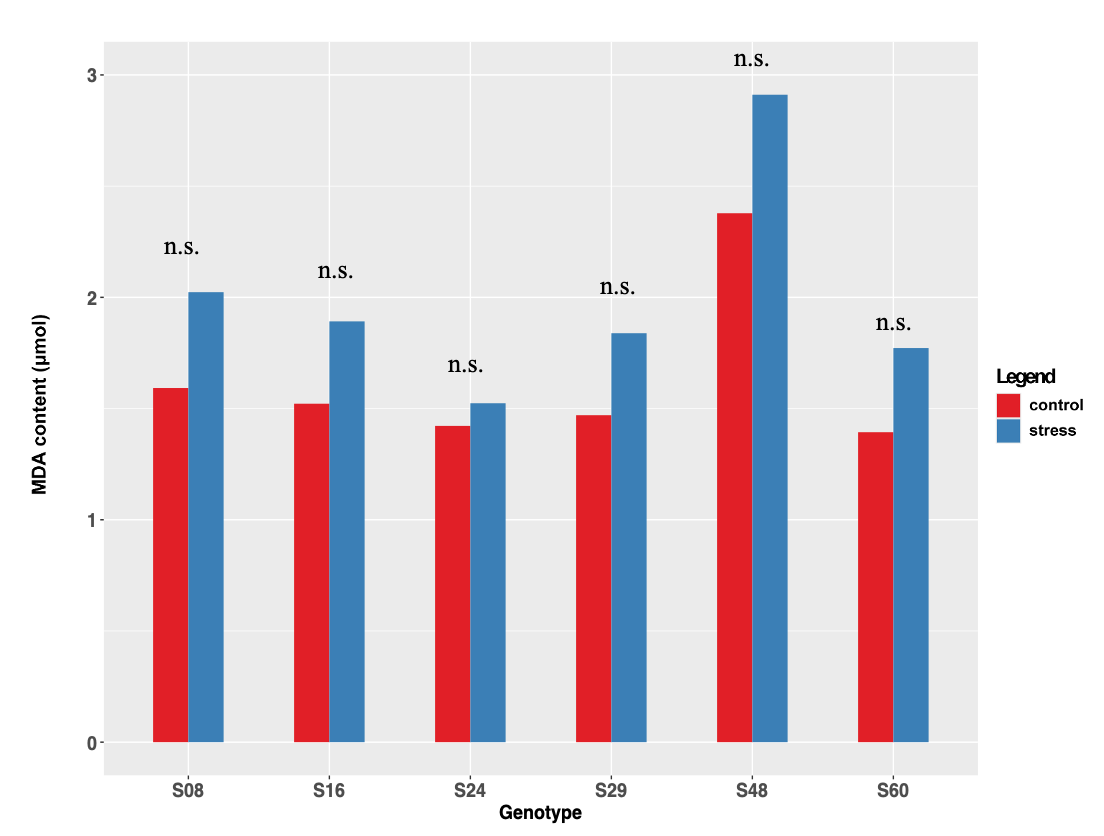
Figure S7.** Effect of water stress on the malondialdehyde (MDA) content in alfalfa genotypes.

**
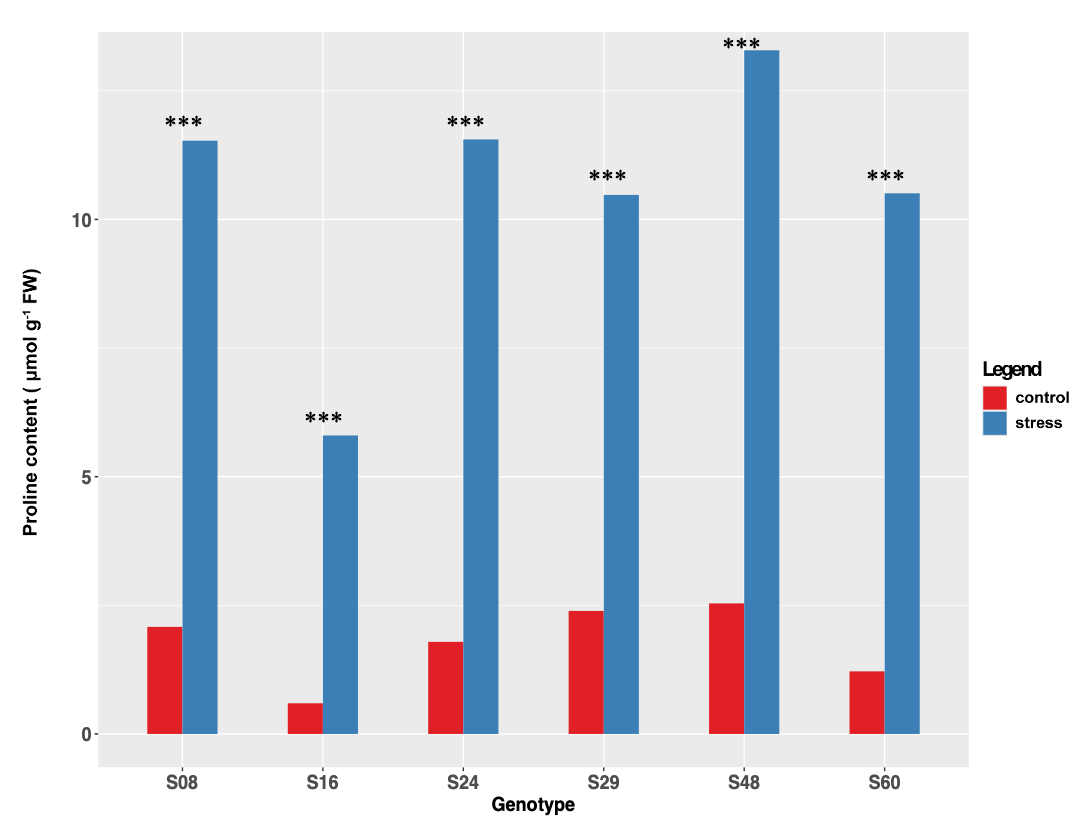
Figure S8.** Effect of water stress on the proline content in alfalfa genotypes.
