# Supplementary material for: Whole genome duplication drives transcriptome reprogramming in response to drought in alfalfa
Source: Plant Cell Rep. 2025 Sep 9;44(10):209. doi: 10.1007/s00299-025-03593-9 (PMC12417302; doi:10.1007/s00299-025-03593-9)
Supplement: Supplementary file 12 — Supplementary file12 (DOCX 335 KB) [file 299_2025_3593_MOESM12_ESM.docx]

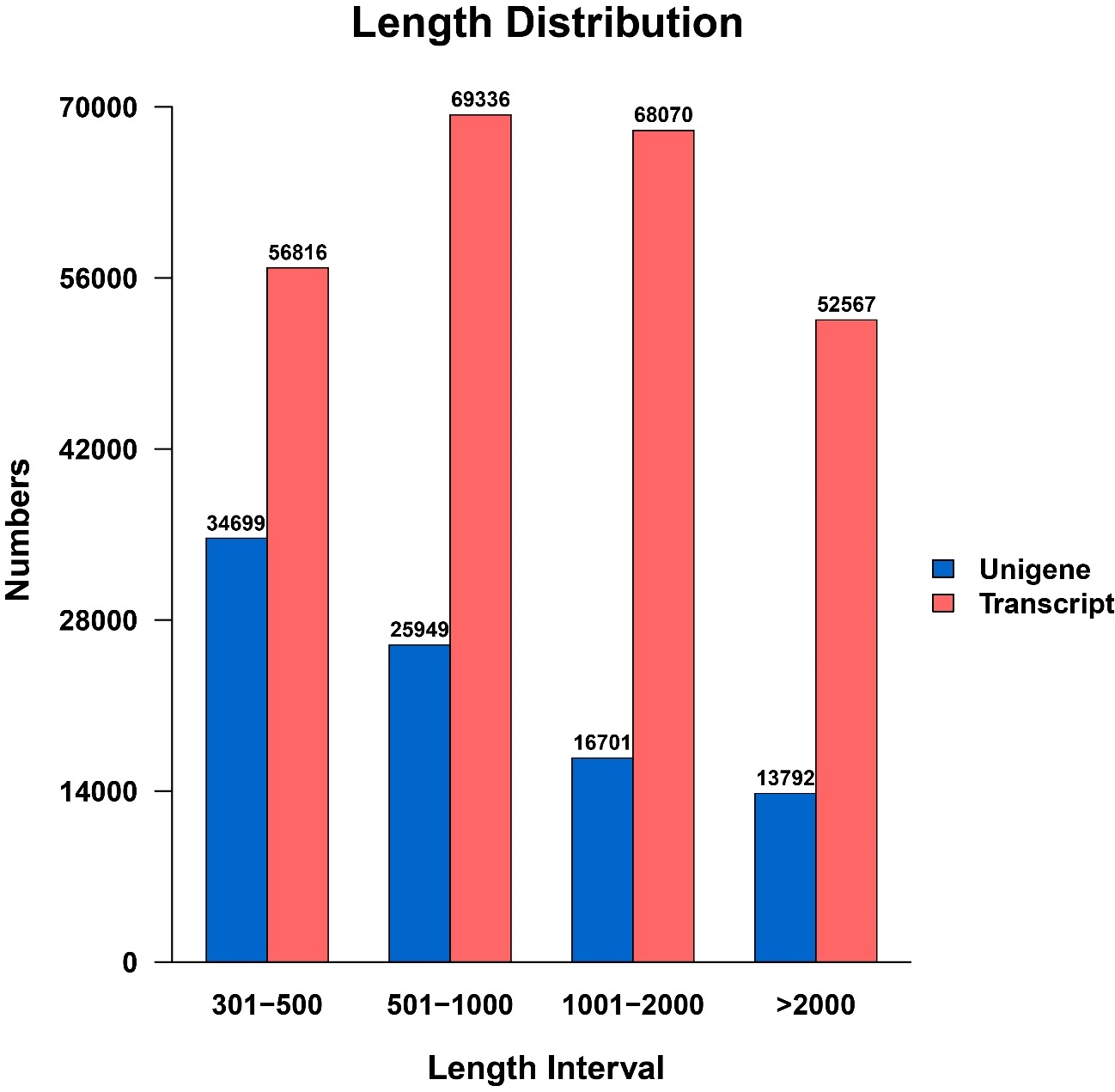


**A**

**Figure S9 A.** Length distribution of transcripts and unigenes obtained from RNA-seq of 2x and 4x alfalfa plants subjected or not to water shortage.


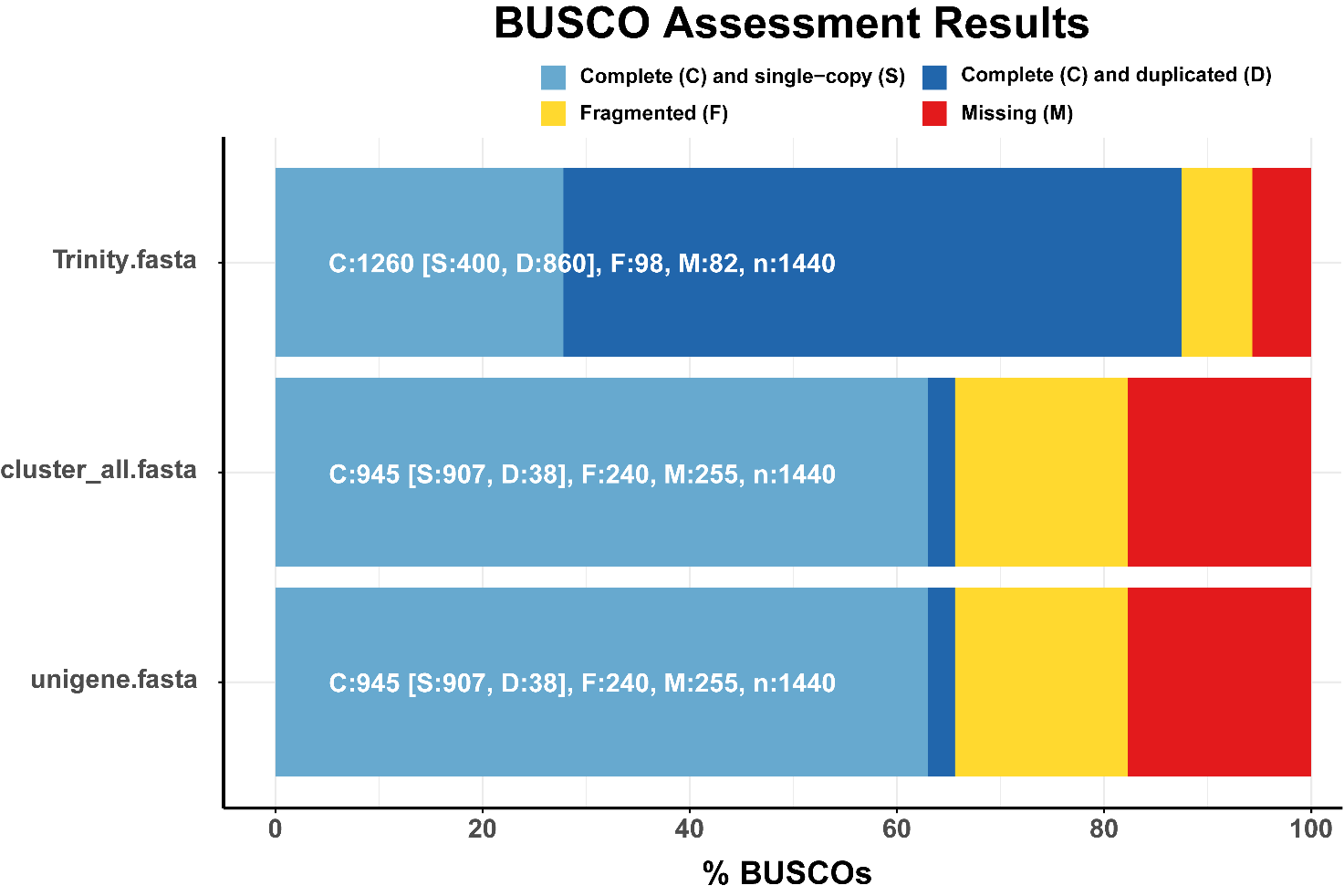


**B**

**Figure S9 B.** BUSCO assessment of transcriptome completeness for the full assembly (Trinity.fasta), the non-redundant transcripts (cluster_all.fasta), and the representative unigenes (unigene.fasta), using BUSCO metrics (C: Complete [D: Duplicated], F: Fragmented, M: Missing, n: number of BUSCOs).
