# Supplementary material for: Whole genome duplication drives transcriptome reprogramming in response to drought in alfalfa
Source: Plant Cell Rep. 2025 Sep 9;44(10):209. doi: 10.1007/s00299-025-03593-9 (PMC12417302; doi:10.1007/s00299-025-03593-9)
Supplement: Supplementary file 13 — Supplementary file13 (DOCX 193 KB) [file 299_2025_3593_MOESM13_ESM.docx]

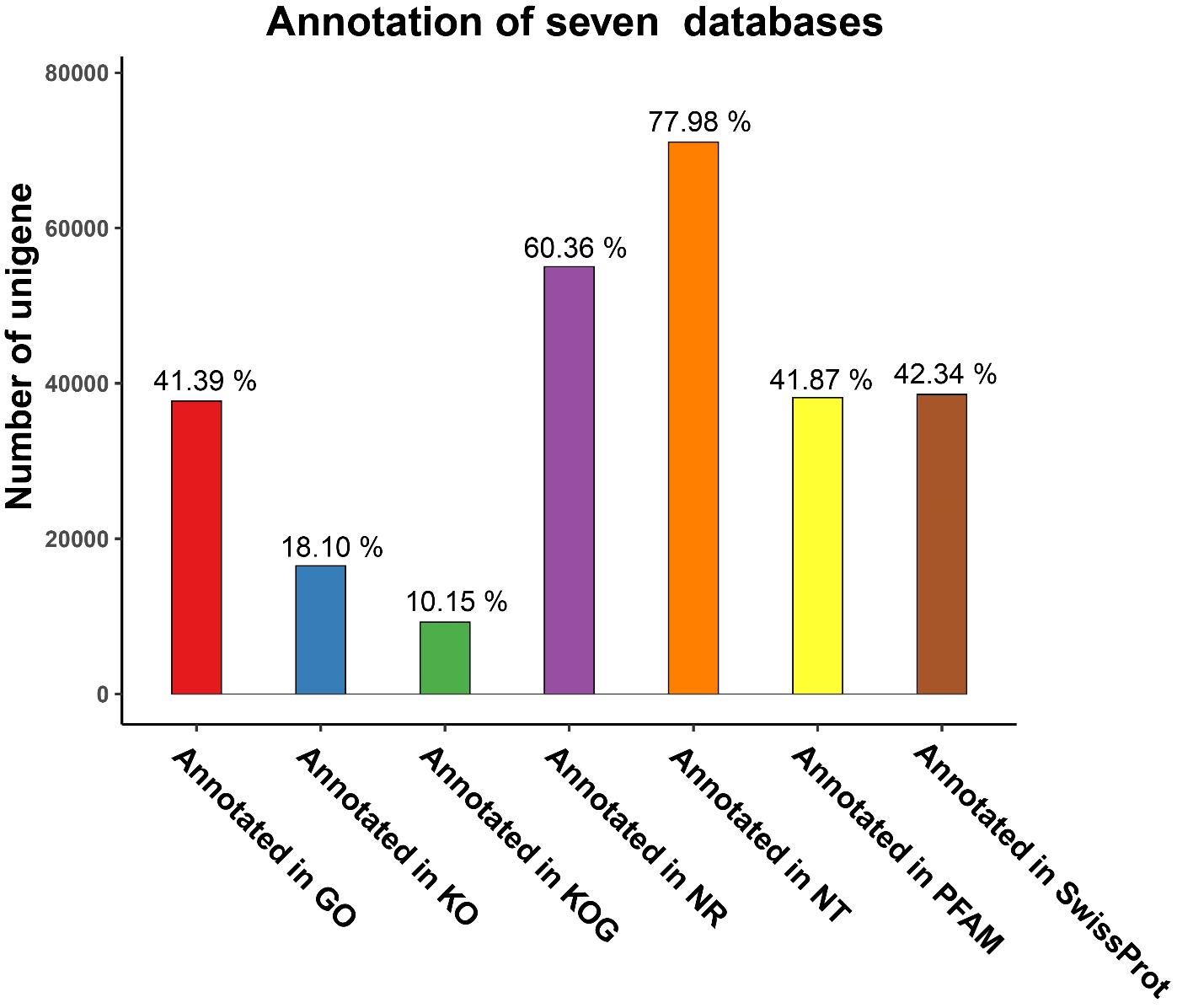


**Figure S10.** Graphical representations of functional annotations of M. sativa leaf transcriptome. The Y-axis shows the number and frequency of annotated unigenes in each database.
