# Supplementary material for: Whole genome duplication drives transcriptome reprogramming in response to drought in alfalfa
Source: Plant Cell Rep. 2025 Sep 9;44(10):209. doi: 10.1007/s00299-025-03593-9 (PMC12417302; doi:10.1007/s00299-025-03593-9)
Supplement: Supplementary file 14 — Supplementary file14 (DOCX 66 KB) [file 299_2025_3593_MOESM14_ESM.docx]

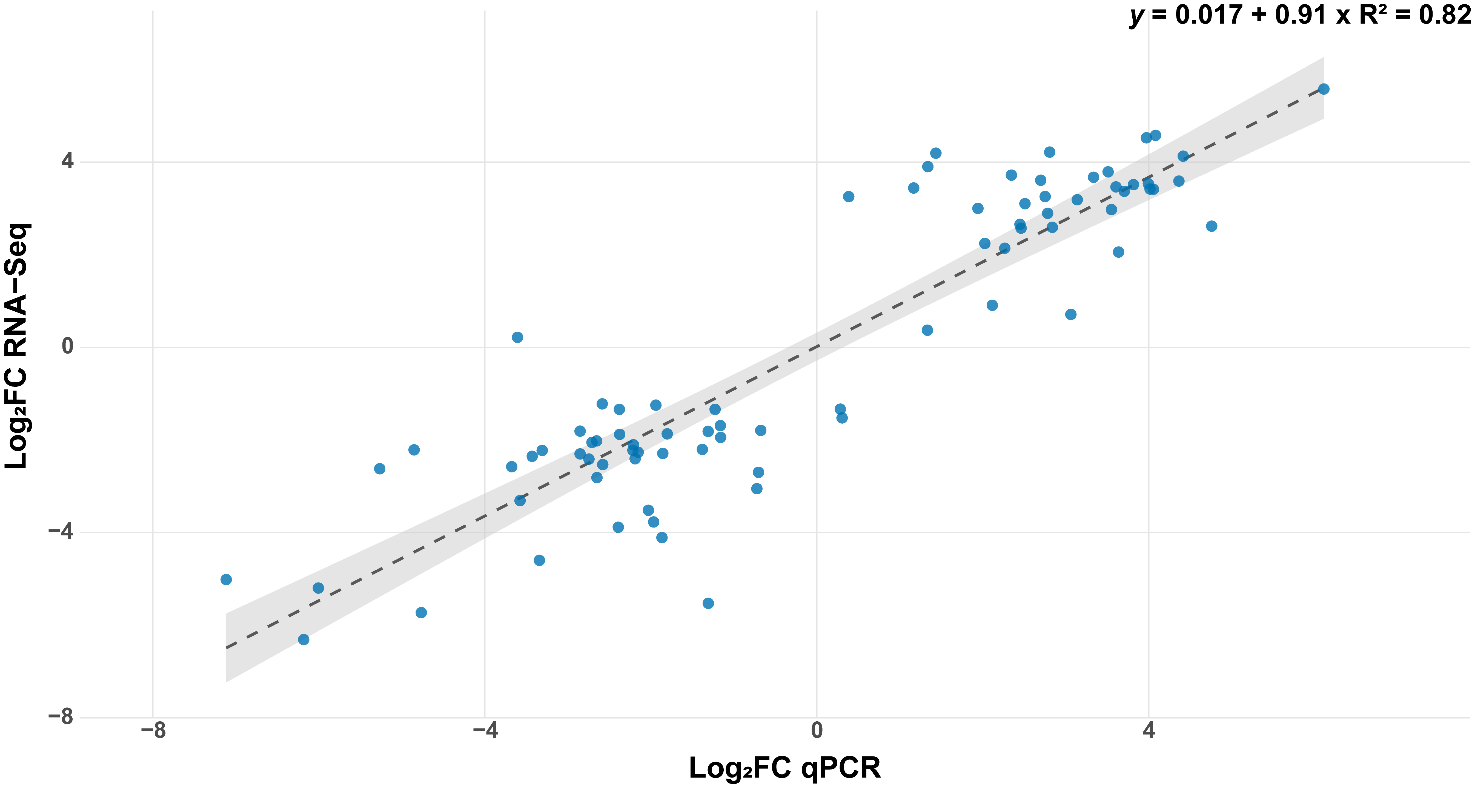


**Figure S11.** Correlation of RNA-seq and RT-qPCR data. Log_2_ fold changes calculated by the 2^−∆∆CT^ method of 14 selected DEGs were plotted against the corresponding data from RNA-seq analysis.
