# Supplementary material for: Whole genome duplication drives transcriptome reprogramming in response to drought in alfalfa
Source: Plant Cell Rep. 2025 Sep 9;44(10):209. doi: 10.1007/s00299-025-03593-9 (PMC12417302; doi:10.1007/s00299-025-03593-9)
Supplement: Supplementary file 15 — Supplementary file15 (DOCX 113 KB) [file 299_2025_3593_MOESM15_ESM.docx]

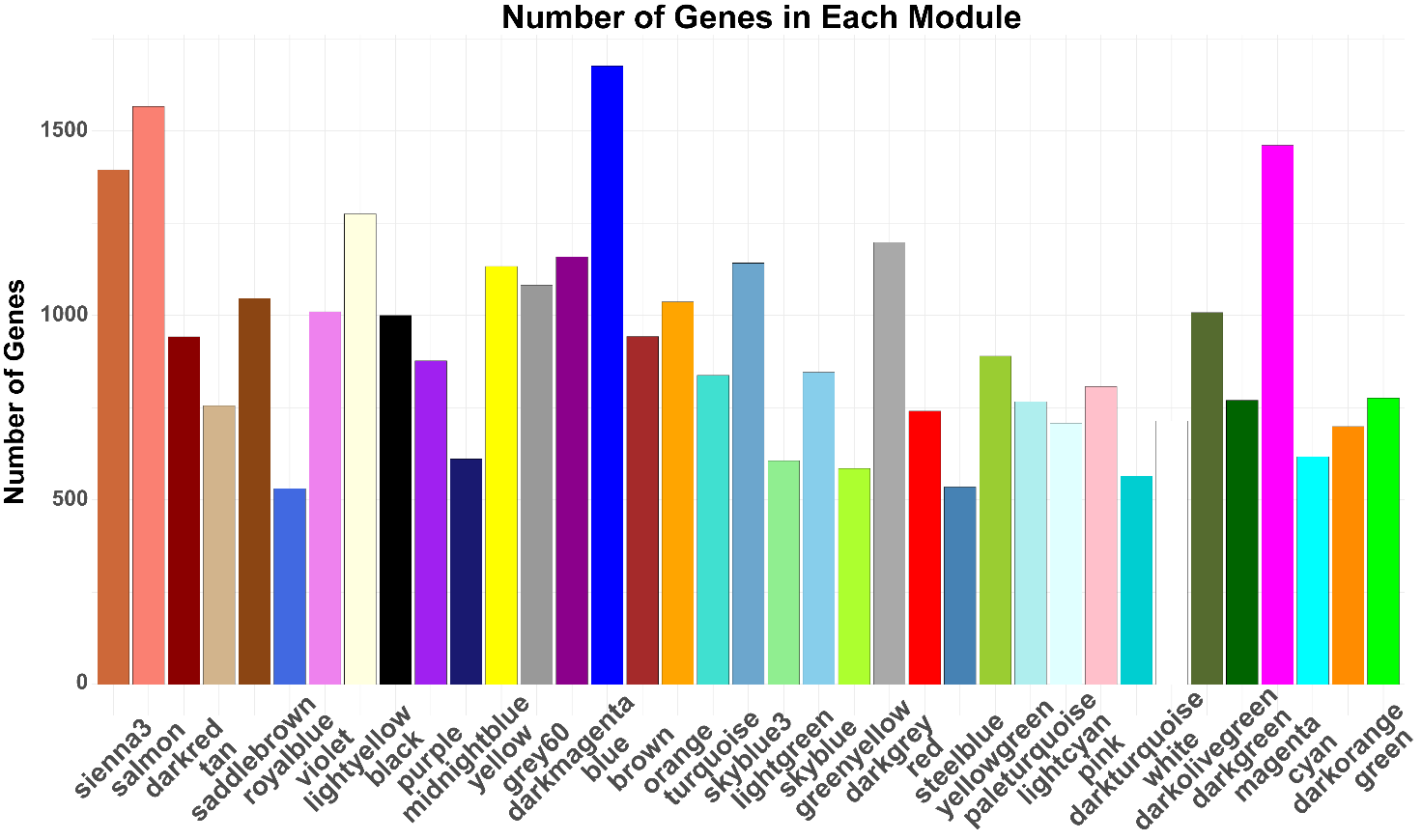


**Figure S12.** WGCNA modules identified by the k-means clustering analysis applied to drought-responsive genes.
